# ITGAM^+^ Extracellular Vesicles Trigger CD8^+^ T Cell Activation via S100a9‐Mediated Metabolic Reprogramming in Acute Kidney Injury

**DOI:** 10.1002/jev2.70404

**Published:** 2026-11-05

**Authors:** Yu‐Qi Fu, Yi‐Lin Zhang, An‐Ran Shen, Ning Li, Jing Song, Yan Bao, Si‐Meng Wang, Ding‐Ding Xue, Min Wu, Bin Wang, Yi Wen, Tao‐Tao Tang, Lin‐Li Lv, Bi‐Cheng Liu

**Affiliations:** ^1^ Institute of Nephrology Jiangsu Provincial Key Laboratory of Renal Injury and Repair Zhongda Hospital Southeast University School of Medicine Nanjing Jiangsu China

**Keywords:** acute kidney injury, ITGAM^+^ EVs, CD8^+^ T cell, metabolic reprogramming

## Abstract

Acute kidney injury (AKI) is a prevalent condition with limited therapeutic options, in which sustained renal injury driven by inflammation remains a central pathological challenge. Here, we identify a specific subset of extracellular vesicles (EVs) as the critical link translating early myeloid inflammation into metabolically fuelled CD8^+^ T cell pathogenicity. Using single‐EV surface proteomics via a proximity barcoding assay in a murine ischemia‐reperfusion injury model, we mapped the renal EV landscape and discovered a myeloid‐derived ITGAM^+^ EV subset with markedly increased abundance after injury. Using a CD63‐PAGFP^ITGAM‐KI^ reporter mouse that genetically enhances ITGAM^+^ EV release, we further validated that these EVs interact with CD8^+^ T cells in the renal lymphatic niche, driving their pro‐inflammatory activation. Proteomic profiling pinpointed S100a9 as the functional cargo on these ITGAM^+^ EVs. Mechanistically, EV‐delivered S100a9 stabilized the mitochondrial aspartate‐glutamate carrier Slc25a12 in recipient CD8^+^ T cells, thereby enhancing glutamate metabolism to fuel a pathogenic transcriptional program. Pharmacological inhibition of S100a9 with tasquinimod, combined with genetic loss of S100a9 in bone marrow‐derived myeloid cells, established the necessity of EV‐associated S100a9 in driving CD8^+^ T cell activation. Our findings establish ITGAM^+^ EVs as essential mediators of pro‐metabolic and pro‐inflammatory signal in AKI and highlight the S100a9–Slc25a12 axis as a promising therapeutic target for intercepting sterile inflammation.

## 1 Introduction

Acute kidney injury (AKI) is a common clinical syndrome associated with high mortality, affecting 10%–15% of hospitalized patients and over 50% of those in intensive care units (Ostermann et al. 2025). Beyond its immediate lethality, AKI is a major risk factor for the subsequent progression of chronic kidney disease (CKD), imposing a substantial long‐term burden on survivors (Zhang et al. 2024). Despite advances in supportive care, clinical management remains limited to non‐specific interventions such as hemodynamic optimization and renal replacement (Levey and James 2017). Targeted therapies for AKI remain challenge due to an incomplete understanding of its pathogenic mechanisms (Yeh et al. 2024, Gaut and Liapis 2020).

Central to its pathogenesis is the release of danger‐associated molecular patterns (DAMPs) from injured renal cells, which ignites a sterile inflammatory cascade driven by innate immune cells. This innate response, in turn, orchestrated the subsequent adaptive immunity, wherein antigen‐presenting cells (APCs) and pathogenic T‐cell responses that exacerbate tissue injury and impede renal recovery (Meng et al. 2025). Previous studies have demonstrated that CD4^+^T cell depletion reduces injury severity in murine ischemia‐reperfusion models, while adoptive transfer of activated CD8^+^T cells exacerbates tubular damage through perforin‐mediated cytotoxicity, establishing adaptive immunity as a key determinant of AKI outcomes (Cao et al. 2016). On the other hand, continuing CD8^+^T cells infiltration induced the peritubular capillary rarefaction which led to the chronic transition of AKI (Jiang et al. 2024). The canonical pathway for T cell activation requires direct cell‐to‐cell contact, wherein professional APCs like dendritic cells process antigens released from damaged tissue and present them to naive T cells, typically within secondary lymphoid organs (Tang and Yiu 2020). However, this classical model does not fully account for the very early infiltration and activation of T cells observed within the renal parenchyma following an acute insult (Lee et al. 2024). How innate immune cells within the injured kidney microenvironment efficiently transmit activating signals to initiate a pathogenic T cell response remains a critical question.

Extracellular vesicles (EVs) are a heterogeneous population of nano‐sized membranous particles released by diverse cell types, functioning as key mediators of intercellular communication within the immune microenvironment (Kalluri 2024). Distinct from soluble factors, EVs protect their encapsulated cargo from degradation, including proteins, lipids, and nucleic acids, enabling targeted delivery to recipient cells and thereby modulating immune cell behaviours (Manno et al. 2025). Indeed, it was recognized early in 1998 that dendritic cell‐derived EVs was capable of presenting MHC class I and II–peptide complexes to prime naïve CD8^+^ and CD4^+^ T cells (Zitvogel et al. 1998). More recently, studies in infectious diseases such as tuberculosis have shown that EVs released from infected macrophages transferred mycobacterial proteins and RNA, which in turn drive a robust Th1 T‐cell response (Wang et al. 2020). These findings highlight a potent, EVs‐mediated mechanism for orchestrating the dialogue between innate and adaptive immunity. However, whether EVs‐mediated communication operates during sterile tissue injury such as AKI remained poorly understood.

A primary obstacle to resolving this uncertainty has been the reliance on traditional bulk EV analysis, which obscure the heterogeneity and is not capable of delineating the complex interplay driven by distinct vesicle subsets. Given that EVs differ dramatically in their biogenesis, surface composition, and cellular origin, resolving EV diversity is essential for identifying the critical EV subpopulation and their precise functions (Buzas 2023). Recent methodological advances enable high resolution analysis of individual vesicles or defined subpopulations. Single‐EV analytical platforms (Zhang et al. 2025), such as nanoflow cytometry, atomic force microscopy, super‑resolution microscopy, and microfluidics‑based particle capture, allow precise characterization of vesicle size, morphology and cargo composition. Among these, the proximity barcoding assay (PBA) (Wu et al. 2019) enabled mapping of surface proteomes on individual EVs via DNA barcoding proximity ligation, thereby revealing functional diversity within EV subsets.

In this study, we employed PBA to delineate the immune microenvironment of ischemia‐reperfusion‐induced AKI at the EV level. Clustering analysis unveiled an ITGAM^+^ EV subpopulation increased remarkably in the post‐ischemic kidney, for which we subsequently elucidated its myeloid‐cell origin and immunoregulatory function. Through engineering a genetic reporter mouse model, the interactions between ITGAM^+^ EVs and CD8^+^T cells were visualized *in vivo*. Mechanistically, ITGAM^+^ EVs deliver their cargo protein S100a9 to CD8^+^ T cells, promoting their activation by promoting mitochondrial metabolism through Slc25a12‐dependent mitochondrial amino acid transport.

## 2 Materials and Methods

### 2.1 Study Design

The objective of this study was to identify pathogenic EV subpopulations in AKI and elucidate the mechanism of action on CD8^+^ T cells. Our approach integrated three parts: (1) An initial discovery phase using PBA (detailed below) on EVs from a murine IRI model to identify key subpopulations. (2) Mechanistic validation study using a custom‐generated CD63‐PAGFP^ITGAM‐KI^ reporter mouse to trace the ITGAM^+^ EV subset *in vivo* and assess its interaction with CD8^+^ T cells, ITGAM^+^ myeloid cells were sorted from S100a9‐knockout mice to investigate the function of ITGAM^+^ EV‐delivered S100a9. (3) Molecular mechanism investigation combines proteomics, super‐resolution imaging, and biochemical assays to define the effect of S100a9 on Slc25a12 expression.

### 2.2 Animal Models

Wild‐type C57BL/6 mice (6–8 weeks) were used to establish unilateral renal ischemia–reperfusion injury (uIRI) for day‐1 AKI modelling (Zhong et al. 2022), after which renal sEVs were isolated for subsequent PBA. Mice were anesthetized with isoflurane and the renal pedicle was clamped for 35 min followed by reperfusion; sham mice underwent identical surgery without clamping. Body temperature was maintained at 37°C throughout the procedure. Renal tissues were collected for subsequent experiments.

To generate a reporter mouse for tracing EVs from ITGAM^+^ cells, we generated a conditional *Itgam‐iCre; CAG‐CD63‐PAGFP* reporter line. Using CRISPR‐Cas9 technology, a *CAG‐LSL‐mCd63‐PA‐GFP‐WPRE‐polyA* cassette was site‐specifically inserted into the murine *H11* locus. These mice were subsequently bred with *Itgam‐iCre* mice, which express Cre recombinase under the control of the myeloid‐specific *Itgam* promoter, to obtain experimental mice. Reporter mice were subjected to uIRI or cisplatin‐induced AKI. For uIRI, the operations were same as depicted above and tissues were harvested at day 1 and day 7 post‐reperfusion. For cisplatin‐induced model, mice received a single intraperitoneal injection of cisplatin (HY‐17394, MedChemExpress,5 mg/kg,). For pharmacological intervention, the S100a9 inhibitor tasquinimod (HY‐10528, MedChemExpress,1 mg/kg/d) was administered via daily oral gavage starting 5 days prior to either IRI or cisplatin induced kidney injury.

For in vivo neutralization of ITGAM, wild‐type C57BL/6J mice received a single intraperitoneal injection of InVivoMAb anti‐mouse/human CD11b antibody (clone M1/70, cat. BE007, Bio X Cell; 500 µg per mouse) 3 h prior to uIRI surgery. Control animals received an equivalent dose of PBS solvent under the same schedule. Kidneys were harvested 24 h after reperfusion for histological, qPCR, immunofluorescence, and flow cytometric analyses.

All animal care and experimental procedures followed the principles of the Declaration, and were in accordance with the Animal Management Rule of the Ministry of Health of the People's Republic of China and approved by the ethics committee of animal experiments at Southeast University.

### 2.3 EV Isolation and Characterization

All EV experiments were conducted in accordance with MISEV guidelines. sEVs were isolated using differential centrifugation. Renal tissues were minced and digested with trypsin at 37°C for 1 h (Zhong et al. 2025), followed by quenching with foetal bovine serum. The homogenate and cell culture supernatant were filtered and subjected to a series of differential centrifugations: first at 2,000 g for 25 min at 4°C to remove cell debris, then at 15,000 g for 30 min at 4°C to pellet large vesicles, and finally at 100,000 g for 2 h at 4°C using a Type 45 Ti rotor (Optima XPN‐100, Beckman Coulter) to pellet EVs. The resulting pellet was resuspended in PBS. Newly isolated sEVs were aliquoted and stored at −80°C for up to one month prior to use.

The size distribution of sEVs were assessed by nanoparticle tracking analysis (NTA) (ZetaView, PMX 110, Particle Metrix, Inning am, Ammersee, Germany). For fluorescent NTA, the same instrument was used with 488 nm filter to detect and quantify PA‐GFP^+^ particles from CD63‐PAGFP^ITGAM‐KI^ mice.

sEV morphology was assessed by transmission electron microscopy (TEM). To validate the surface localization of specific cargo, immunoelectron microscopy (Immuno‐EM) was conducted by incubating grid‐adsorbed sEVs with anti‐S100a9 antibody (26992‐1‐AP, Proteintech) followed by a colloidal gold‐conjugated secondary antibody prior to negative staining Furthermore, the expression of classic sEV markers, including negative marker GM130 (sc‐55590, Santa Cruz), positive marker CD63 (sc‐5275, Santa Cruz), and CD9 (84801‐5‐RR, Proteintech), was characterized by Western blot analysis.

### 2.4 Single‐vesicle Analysis With PBA

To achieve a high‐throughput analysis of the surface proteome on individual EVs, we employed a PBA (Wu et al. 2019). Briefly, renal tissue‐derived EVs (5 µL) were immobilized on CTB‐coated 96‐well plates via GM1 ganglioside binding and incubated for 1 h at room temperature. Following three PBST washes, captured EVs were probed with DNA‐labelled antibodies from the ExoSeek Panel 260 (Secretech, Shanghai, China) for 2 h. Following washes to remove unbound probes, rolling circle amplification (RCA) products were added. An annealing and extension reaction then ligated the antibody barcodes to their complementary sequences on the RCA products, thus assigning a unique barcode to each EV. The resulting DNA library was amplified via PCR, and the amplicons were sequenced using a PE150 strategy on an Illumina NovaSeq S4 platform. Finally, the sequencing data was processed through the EVisualizer decoding package (Secretech) to demultiplex reads, quantify protein signals for each individual EV, and generate a comprehensive digital protein map of the EV population.

### 2.5 ITGAM^+^ EV Immunomagnetic Sorting

ITGAM^+^ EVs were isolated using an immunomagnetic affinity‐based EV isolation kit (EVlent Magnetic kit, Evlixir Biotechnology, Nanjing, China) following the manufacturer's instructions. Briefly, EV samples were diluted 1:4 in Incubation Buffer I and incubated with EVlent magnetic beads for 1 h at room temperature with gentle rotation. After magnetic separation, bead‐bound EVs were washed sequentially with Incubation Buffer II and Washing Buffer to remove unbound material. For ITGAM‐specific isolation, EVlent beads were pre‐coupled with anti‐ITGAM antibodies prior to sample incubation, enabling selective capture of ITGAM^+^ EV subsets.

Downstream processing was tailored to experimental applications according to kit instructions. For proteomic analysis, bead‐bound EVs were lysed directly in EV lysis buffer (EV02‐08‐01, Evlixir Biotechnology, Nanjing, China). For Western blotting, beads were mixed with sample loading buffer and heated at 95°C for 5 min; the supernatant obtained after magnetic separation was subjected to SDS‐PAGE. For NTA or TEM, ITGAM^+^ EVs were eluted by vortexing beads in Elution Buffer for 10 min, followed by magnetic separation; eluates from two rounds were combined for analysis.

### 2.6 Primary CD8^+^ T Cell Isolation, Culture, and siRNA‐Transfection

Spleens were harvested from C57BL/6J mice and mechanically dissociated to create single‐cell suspensions. CD8^+^ T cells were isolated by negative selection using a Mouse CD8^+^T Cell Isolation Kit (19853, Stemcell) according to the manufacturer's protocol. Isolated CD8^+^ T cells were cultured in IMDM medium (Gibco) supplemented with 10% FBS (Gibco), 1% penicillin‐streptomycin, 50 µM β‐mercaptoethanol and IL‐2(212‐12, Pepretech,10 ng/mL) and stimulated with anti‐CD3 (2678859, Invitrogen,5 µg/mL) and anti‐CD28 (2918513, Invitrogen,2 µg/mL).

For EV intervention assays, cells were treated with sEVs isolated from the indicated groups; to ensure experimental consistency, sEV dosage was normalized by total protein quantity across all conditions. Pharmacological inhibition was performed using the S100a9 inhibitor Tasquinimod (HY‐10528, MedChemExpress, 5 µM). To evaluate the efficacy of vesicular versus soluble delivery, cells were treated with recombinant murine S100a9 (HY‐P74583, MedChemExpress) at a concentration determined by ELISA to be equivalent to the S100a9 abundance present on ITGAM^+^ sEVs.

For siRNA‐mediated knockdown of *Slc25a12*, cells were transfected using the ProteanFect Max Mouse Immunocyte Transfection Kit (Nanoportal Biotech, PT03) according to the manufacturer's protocol. A custom‐synthesized siRNA duplex targeting *Slc25a12* (sense: 5'‐GGUUGAUCUCCUAUCAAGATT‐3'; antisense: 5'‐UCUUGAUAGGAGAUCAACCTT‐3') or a non‐targeting control siRNA were used. Transfected cells were cultured for 72 h before being used in subsequent experiments.

### 2.7 Primary ITGAM^+^ Myeloid Cell Isolation and Culture

Global S100a9 knockout mice (strain: C57BL/6JSmo‐S100a9^em1Smoc^; catalog no. NM‐KO‐190143; Shanghai Model Organisms Center, Inc.) were purchased and maintained under specific‐pathogen‐free (SPF) conditions identical to those of wild‐type controls. Age‐ and sex‐matched littermate WT C57BL/6J mice served as control animals. Bone marrow and spleen were harvested from S100a9 KO mice and littermate WT controls. Bone marrow cells were flushed from femurs and tibiae with cold PBS, and splenocytes were obtained by mechanical dissociation through 70 µm strainers; both were subjected to red blood cell lysis. ITGAM^+^ myeloid cells were positively selected using MicroBeads (human and mouse, cat. 130‐049‐601, Miltenyi Biotec) on LS columns according to the manufacturer's instructions. Sorting purity was verified by flow cytometry. Purified ITGAM^+^ cells were seeded into T25 flasks in DMEM supplemented with 10% exosome‐depleted FBS (Gibco), 1% penicillin‐streptomycin, and recombinant murine M‐CSF (PeproTech, 20 ng/mL). On day 5, conditioned media were then collected and processed for sEV isolation as described above.

### 2.8 Nano‐Flow Cytometry

sEVs were characterized using nano flow cytometry (Apogee A60 Micro Plus, Apogee Flow). The instrument was calibrated following a two‐step procedure: Apogee 1524 microspheres were used for optical alignment, followed by Apogee 1527 particles for performance tracking. Prior to sample analysis, sheath fluid and PBS were run as controls to confirm the absence of background particles. For immunolabeling, isolated sEVs were incubated with a primary antibody: FITC anti‐mouse CD11b (ITGAM) (101206, Biolegend, 0.5 mg/mL), Brilliant Violet 421 TM anti‐mouse F4/80 (123131, Biolegend, 50 µg/mL), PE anti‐mouse LY6G (PE‐65140, Proteintech, 0.2 mg/ml) CD11c (117310, Biolegend, 0.2 mg/mL) for 1 h at 37°C, followed by a secondary antibody for 30 min at 37°C. All samples were diluted to a final volume of 250 µL with PBS for acquisition. An unstained control, consisting of sEVs without antibody labelling, was included. Data analysis was restricted to particles with a diameter of 80–100 nm, as determined by standard microspheres.

### 2.9 Live Kidney Section Imaging

For real‐time visualization of EV‐T cell interactions, live kidney slice imaging was performed using CD63‐PAGFP^ITGAM/KI^ reporter mice as previously established (He et al. 2024). First, kidney sections were prepared from mice 2 h after intravenous injection of a fluorescent antibody: eFluor anti‐moLYVE1 (2943441, Invitrogen); Alexa Flour594 anti‐CD8a (100758, Biolegend) following unilateral renal ischemia‐reperfusion surgery. The kidneys were immediately harvested and sectioned into 25 µm slices using a vibratome (Leica VT1200S). These sections were maintained in a humidified chamber at 37°C for 2 h before imaging. A confocal microscope (Zeiss Elyra 7) configured for four‐dimensional acquisition was used. The acquisition settings were as follows: Z‐stack range, 0–25 µm; Z‐step, 1 µm; time interval, 5 min; total duration, 100 min. Subsequent image processing and analysis were performed using Arivis software.

### 2.10 Super‐Resolution Imaging

All dSTORM images of EVs were acquired on Oxford Nanoimaging System (ONI, Oxford). For dSTORM imaging of EVs, samples derived from mouse tissue or cells were captured onto Assay Chips and incubated with primary antibodies against ITGAM (sc‐1186, Santa Cruz) and S100a9 (26992‐1‐AP, Proteintech) following the protocol of EV Profiler Kit (EV‐MAN‐1.0, ONI, Oxford). Secondary antibodies were incubated for another 1 h. Finally, the chips were washed and applied with image buffer for immediate imaging. Channel mapping was first performed using Fluorescent Microsphere (0.1 µm TetraSpeck, Thermofisher). sEVs images were acquired using NimOS software (version 1.19, ONI). Images were analysed with ONI online platform CODI (https://alto.codi.bio/) using EV profiling App as described (Lucotti et al. 2025).

For dSTORM imaging of CD8^+^T cells, co‐incubation with ITGAM^+^ sEV^IRI^ (pre‐stained with PKH67) and stained based on ONI Mitochondria Staining Protocol. Cells were incubated in fixation buffer (4% PFA with 0.2% glutaraldehyde in PBS) followed by reducing buffer (0.1% w/v. Mix 10 mg of NaBH_4_ into 10 mL of PBS) incubation. Then cells were washed with PBS by centrifugation. Then, block cells with the blocking buffer followed by incubation with primary antibodies anti‐TOMM20 (ab186735, abcam), anti‐Slc25a12 (67467‐1‐Ig, Proteintech) and membrane dye PKH26 for 1 h at RT. Secondary antibodies conjugated with Cy5 or Alexa Fluor 488 were incubated for 1 h at RT. After wash, the cells were transferred onto 1.5H coverslip (10812, Ibidi) and perform a post fixation with 4% PFA in PBS. Finally, apply image buffer (ONI, Oxford) on cell surface and image immediately. Before imaging, channel mapping was performed as previously. Images were taken with Oxford Nanoimaging System (NanoimagerTM Software, Version: 1.19, ONI) and analysed with CODI online platform (https://alto.codi.bio/) with the Cluster & Counting application.

### 2.11 EV Uptake and Live‐Cell Imaging

For EV uptake experiments, ITGAM^+^ or ITGAM^−^sEV were labelled with PKH67 Exosome Staining Kit (C3635S, Beyotime) according to the manufacturer's protocol. Primary CD8^+^ T cells were seeded onto Poly‐L‐Lysine Solution (PLL) coated 8‐well chamber slide (80826, Ibidi) and incubated with PKH67‐labeled sEVs (10 µg/mL) overnight. Before imaging, CD8^+^ T cells were incubated with MitoTracker Deep Red FM (C1032, Beyotime) for 30 min at 37°C to label mitochondria. Live‐cell imaging was performed using Nanoimager to monitor the internalization of EVs and their colocalization with mitochondria over time. The temperature was set to 37°C and performed channel mapping afterwards. Images were captured simultaneously in the 640 and 488 nm channels at 12% each for 1000 frames, with a 5‐min interval and the process lasted for 40 mins. The images were analysed with Oxford Nanoimaging System (NanoimagerTM Software, Version: 1.19, ONI).

### 2.12 Proteomic Analysis

For single‐vesicle surface proteome profiling, a PBAwas employed using the ExoSeek panel260 kit (Secretech, Shenzhen, China). This technique utilizes a panel of 260 DNA‐barcoded antibodies to profile surface proteins on individual EVs. The resulting barcode libraries were subjected to high‐throughput sequencing on an Illumina NovaSeq platform. Data was subsequently decoded and analysed with the EVisualizer software (v1.0) to quantify protein expression, co‐localization patterns, and define distinct EV subpopulations.

For ITGAM^+^EV bulk proteomic analysis, sEV samples were submitted to EVLiXiR Biotech (Nanjing, China). Proteins were denatured, reduced, and alkylated prior to overnight digestion with trypsin. The resulting peptide mixtures were desalted and analysed by liquid chromatography‐mass spectrometry (LC‐MS) using a Q Exactive HF‐X instrument (ThermoFisher, USA). Acquired spectra were subsequently processed and analysed using Proteome Discoverer (v. 1.3).

Immunoprecipitation‐Mass Spectrometry (IP‐MS): Lysates from ITGAM^+^ sEV‐treated CD8^+^ T cells were incubated with an anti‐S100a9 antibody (26992‐1‐AP, Proteintech) or an isotype control IgG (30000‐0‐AP, Proteintech), followed by incubation with Protein A/G magnetic beads. After washing, bound proteins were eluted and analysed by LC‐MS/MS as described above to identify S100a9‐interacting partners.

### 2.13 Molecular Docking

Protein–protein docking between S100a9 and Slc25a12 was performed using GRAMM (http://gram.compbio.ku.edu/), a rigid‐body docking algorithm. Murine amino acid sequences of S100a9 and Slc25a12 were retrieved from UniProtKB, and three‐dimensional structural models were generated by SWISS‐MODEL homology modelling using template PDB IDs 5I8N (S100a9) and 4P60 (Slc25a12). The resulting PDB files were submitted to GRAMM for docking, generating ten candidate complexes ranked by predicted binding score. The top‐ranked pose was selected for downstream analysis. Interface parameters, including buried surface area and solvation free energy gain (ΔiG), were calculated with PDBePISA, and the complex was visualized in PyMOL.

### 2.14 Statistical Analysis

Statistical analyses were carried out by GraphPad Prism 7 (GraphPad, La Jolla, CA, USA). Data were presented as mean ± SD. For data comparation among groups, Two‐tailed unpaired *t*‐test and One‐way ANOVA was applied. p < 0.05 was considered significant.

## 3 Results

### 3.1 Profile of EVs‐Associated Immune Microenvironment After IRI‐AKI

To resolve the composition of the EV‐associated microenvironment in AKI, PBA was applied that enables surface proteome profiling at the single‐vesicle level. AKI was induced by unilateral ischemia‐reperfusion injury (IRI). Histologically, IRI kidneys displayed typical features of acute tubular injury, including tubular dilation, brush border effacement, and the formation of intratubular casts, accompanied by interstitial inflammatory cell infiltration. This was quantitatively confirmed by increased tubular injury scores and significantly elevated inflammatory cytokine IL‐1β and IL‐6 compared to sham controls (Figure 1A–D).

**FIGURE 1:**
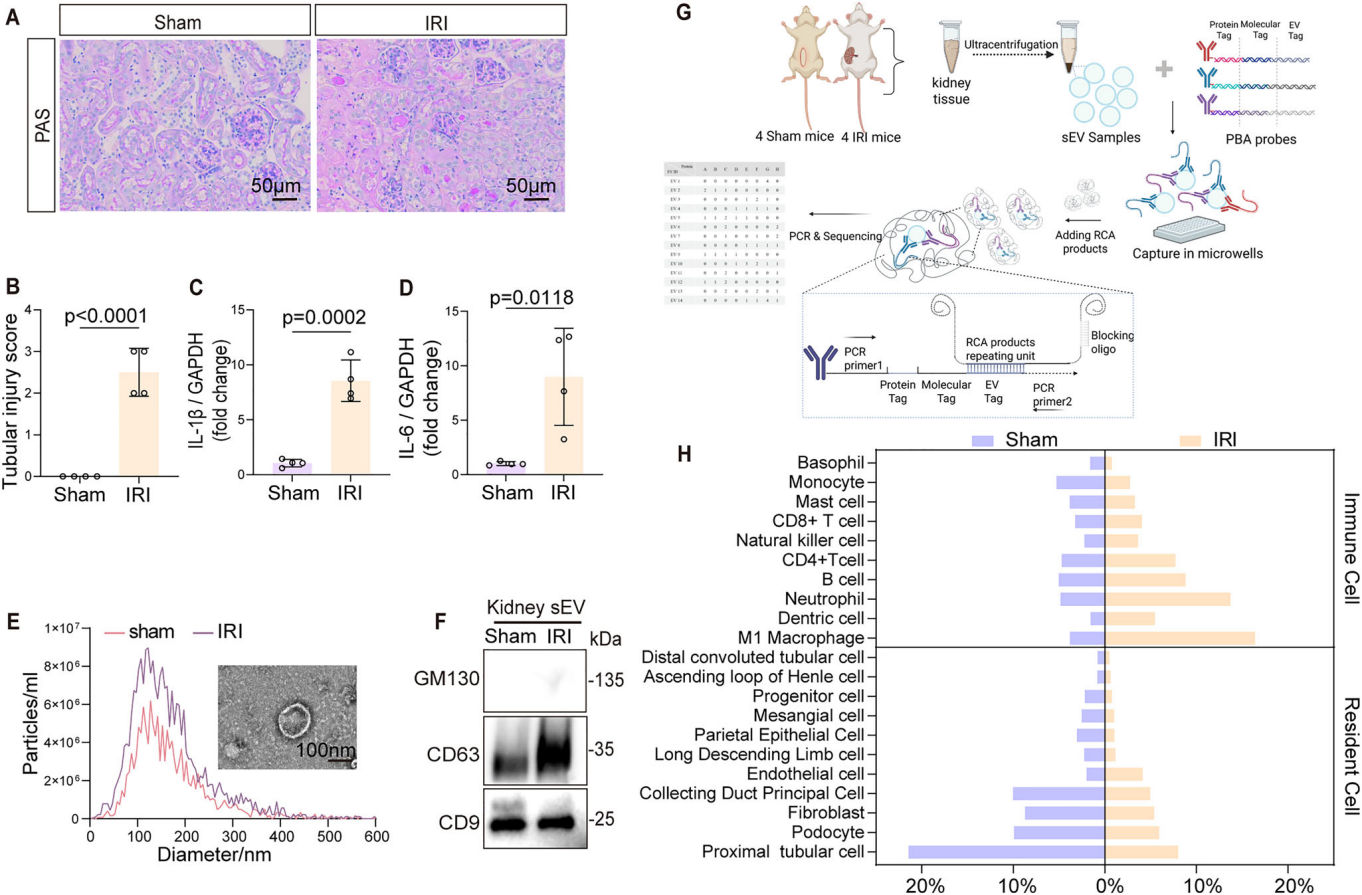
Landscape of the EV‐associated renal immune microenvironment after AKI. (A) Representative images of periodic acid–Schiff (PAS) staining of kidney sections. Bars = 50 µm (B) Quantification of tubular injury score (*n* = 4). (C) IL‐1β and (D) IL‐6 mRNA expression quantified by RT‐qPCR (*n* = 4). (E) Particle size distribution of kidney sEV assessed by Nanoparticle Tracking Analysis (NTA) (left) and morphology examined by transmission electron microscopy (TEM) (right). Scale bar, 100 nm; (F) Western blot analysis of EV markers (CD63, CD9) and non‐EV marker GM130. (G) Schematic illustration of the proximity barcoding assay (PBA) workflow (*n* = 4). (H) Proportional distribution (% of total renal sEVs) of EVs assigned to each cellular origin based on PBA analysis. Data are presented as mean ± SD. Statistical significance was determined by a two‐tailed unpaired Student's *t*‐test.

EVs were then isolated from the kidney tissues which were validated by the characteristic cup‐shaped morphology, size distribution, and enrichment of EV markers (Figure 1E,F). The PBA was designed using a 264 membrane‐associated antibody panel targeting a broad spectrum of surface proteins, including lineage markers for various immune and renal cells, adhesion molecules, chemokine receptors, and immune‐regulatory proteins (Table S1). The reproducibility of the assay was validated with correlation analysis showing high intra‐group consistency and clear separation between the IRI and sham biological replicates (Figure S1A). Interestingly, protein amounts and total EV counts as detected by PBA showed no significant difference between IRI and sham groups. On the contrary, the protein per EV ratio, defined as the total protein barcode counts divided by the total EV counts for a given sample was significantly elevated in the IRI group (Figure S1B), suggesting kidney EV composition changed with injury.

To profile EV associated microenvironment, EV was sourced by cellular mapping through calculating the proportion of EVs expressing canonical markers of immune and renal intrinsic cell (defined by reference to CellMarker 2.0 database; Table S2). Impressively, we identified remodelling of EV profile characterized by a marked increase in immune‐cell derived EVs in the IRI group, with remarkable proportion from M1 macrophage, dendritic cell and neutrophil. EVs derived from effector lymphocytes (CD4^+^T, CD8^+^T, and B cells) displayed elevated proportions as well. Conversely, the proportion of EVs from most renal resident cell populations declined (Figure 1G,H). Overall, these findings establish that the post‐injury renal EV‐associated microenvironment displayed a change of EV profile characterized with remarkable increased proportion of EVs originating from infiltrating immune cells, especially myeloid immune cells.

### 3.2 A Distinct ITGAM^+^sEV Population of Myeloid Origin Expands in IRI‐Kidney

Leveraging the unique capability of PBA technology to generate a single‐EV resolution matrix of surface protein expression, unsupervised clustering of EV subpopulation was performed using the Seurat package. This analysis successfully identified 8 major distinct subgroups, each defined by one or two surface markers. While EV subpopulations expressing the tubular damage marker HAVCR1 were abundantly present, reflecting the established pathology of IRI, a distinct population of EVs characterized exclusively by the myeloid cell marker ITGAM emerged as one of the most dominant subpopulations. The remaining subclusters were characterized by markers involved in immune regulation, including the adhesion integrin ITGAV, the chemokine receptor for the antigen‐cross‐presenting dendritic cell subset XCR1, and the immunomodulatory ectoenzyme NT5E. Another group of markers reflected the status of renal resident cells, including the endothelial glycoprotein EMCN, the tubular reabsorption protein CUBN, and the adhesion molecules CDH4 and CADM3, which signify epithelial damage or reorganization (Figure 2A–C). Therefore, the principal EV clusters were primarily related to renal resident cell injury and ongoing immune responses.

**FIGURE 2:**
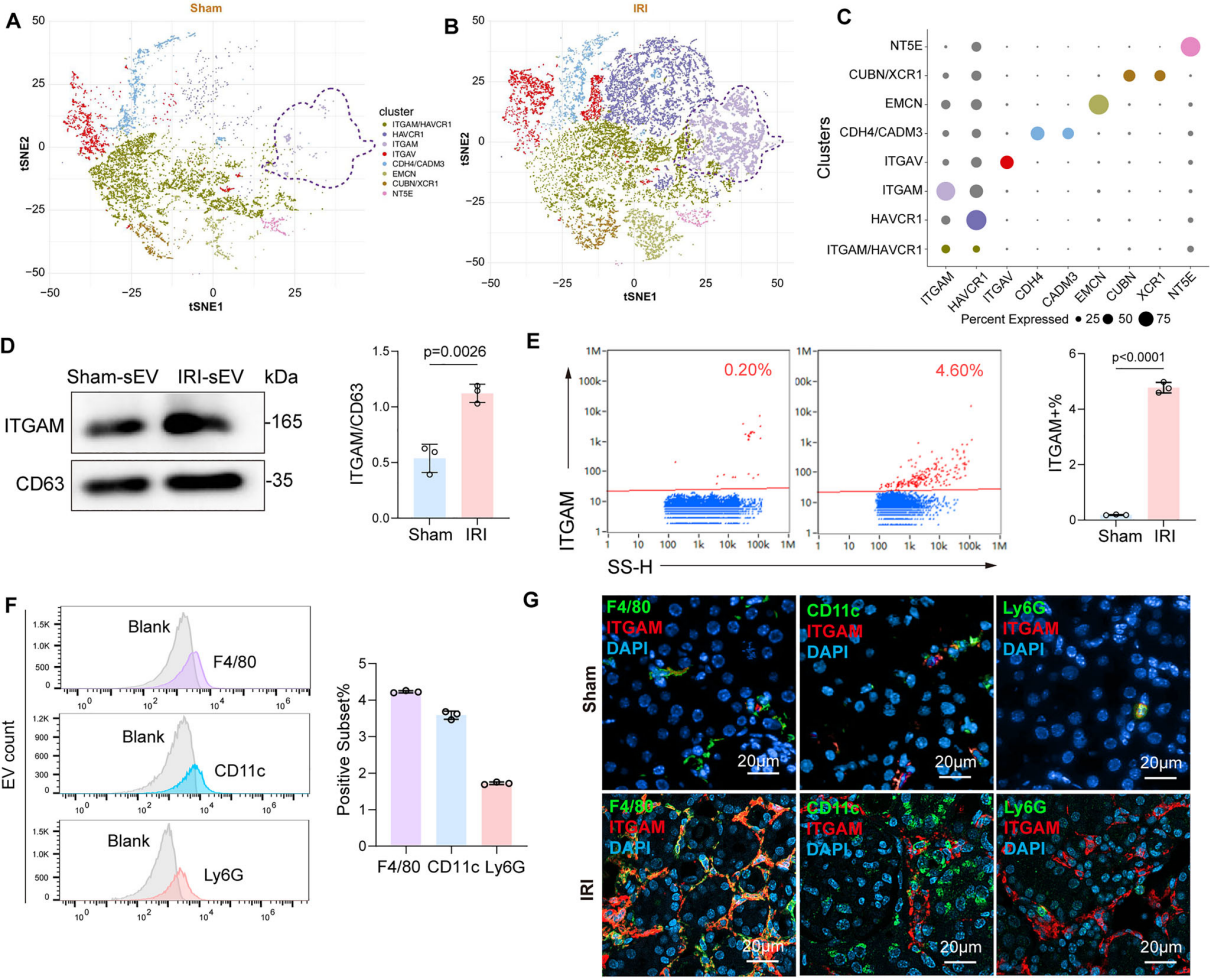
Single‐EV analysis identifies ITGAM^+^ sEV as a dominant, myeloid‐derived subpopulation in IRI kidney. (A, B) t‐distributed stochastic neighbour embedding (tSNE) plots of single EVs from (A) sham and (B) IRI kidneys. (C) Dot plot of marker proteins defining each EV clusters. (D) Western blot and (E) nanoflow cytometry (nanoFCM) analysis of the ITGAM^+^ sEV (*n* = 4). (F) Cellular origin deconvolution of ITGAM^+^ sEV in IRI mice by nanoFCM (*n* = 3). (G) Representative immunofluorescence images of ITGAM (red) and myeloid cell markers (green) in kidney sections. Scale bars, 20 µm. Data are presented as mean ± SD. Statistical significance for (D, E) was determined by a two‐tailed unpaired Student's *t*‐test. For (F), one‐way ANOVA followed by Tukey's multiple comparisons test was performed.

Based on the remarkable increase of myeloid cell derived ITGAM^+^ subpopulation, we proceeded to validate its presence and cellular origin. As expected, an increase in ITGAM expression was detected in EVs from the IRI group compared to the sham group (Figure 2D). Consistent results were obtained by nanoparticle flow cytometry, with an elevated proportion of ITGAM^+^sEV detected in IRI‐kidney (Figure 2E). Deconvolution of their cellular source identified that ITGAM^+^sEVs were primarily derived from macrophages (F4/80) and dendritic cells (CD11c), with a less contribution from neutrophils (Ly6G) (Figure 2F), among the total kidney EV populations. Tissue staining revealed increased colocalization of ITGAM with F4/80, CD11c, and Ly6G+ myeloid cells in IRI‐kidneys compared with sham controls, confirming the increased infiltration of source cells (Figure 2G). Further supporting this myeloid origin, analysis of a public single‐cell RNA sequencing dataset (GSE267242) from an analogous IRI model confirmed robust *Itgam* transcript expression specifically within neutrophil, macrophage, and dendritic cell populations in the injured kidney (Figure S2). Therefore, these findings establish that IRI‐AKI induces the expansion of a distinct ITGAM^+^sEV population predominantly originating from infiltrating myeloid cells.

### 3.3 ITGAM^+^ sEV Drives CD8^+^T Cell Activation as Identified by Proteomics Analysis

Further, a differential proteomic analysis of total kidney EVs was performed to explore the function of altered EV landscape. This analysis revealed a distinct set of 14 upregulated proteins in EVs from IRI versus sham kidneys (Figure 3A). Functionally, these proteins signature was predominantly associated with immune cell adhesion (ITGAM, ITGAL, ITGA6, ITGAV), endothelial adhesion (EMCN), and neutrophil cytotoxic response (LY6G, MPO). The profile also included mediators of T‐cell crosstalk (CD5, CD36, XCR1), markers of inflammatory cascade, tissue damage (HAVCR1, AQP1) and the metabolic hormone ADIPOQ. Gene Ontology Biological Process enrichment revealed T cell‐mediated cytotoxicity, positive regulation of cell killing and antigen processing and presentation of endogenous antigen (MHC class I pathways), supporting a tight association between the differential EV signature and CD8^+^ T cell effector programs (Figure 3B).

**FIGURE 3:**
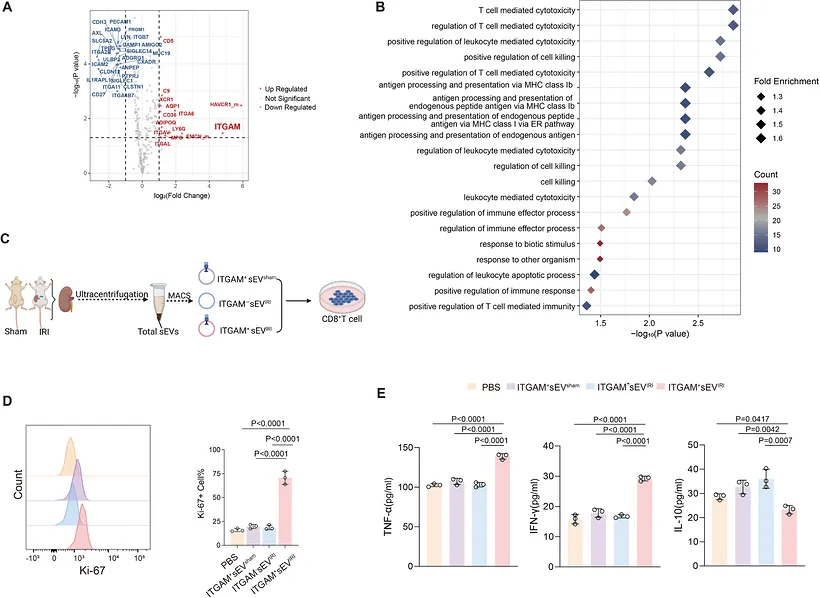
Proteomic characterization reveals ITGAM^+^ sEV promote CD8^+^ T cell proliferation and activation. (A) Volcano plot showing differentially expressed proteins in kidney sEV from IRI versus sham mice. (B) The top 20 enriched Gene Ontology (GO) biological process pathways. (C) Schematic overview illustrating the evaluation of the effects of ITGAM^+^ sEV on CD8^+^ T cell assay. (D) Ki‐67 histogram showing CD8^+^ T cell proliferation at 24 h treated with ITGAM^+^ sEV from sham mice (ITGAM^+^ sEV^sham^), ITGAM‐negative sEV from IRI mice (ITGAM^−^sEV^IRI^), and ITGAM^+^ sEV from IRI mice (ITGAM^+^sEV^IRI^) (*n* = 3). (E) ELISA quantification of TNF‐α, IFN‐γ, and IL‐10 secretion (*n* = 3). Data are presented as mean ± SD. Statistical significance for (D,E) was determined using one‐way ANOVA followed by Tukey's multiple comparisons test.

To investigate the biological function of ITGAM^+^sEV, this EV subcluster was isolated with magnetic beads and co‐cultured with CD8^+^T cells isolated from the mice spleens (Figure 3C) Impressively, ITGAM^+^sEV purified from IRI kidneys (ITGAM^+^sEV^IRI^) but not their ITGAM^−^ counterparts (ITGAM^−^sEV^IRI^) or ITGAM^+^sEV from sham controls (ITGAM^+^sEV^sham^), enhanced CD8^+^T cell proliferation (Figure 3D). In addition, ITGAM^+^sEV^IRI^ promoted the secretion of pro‐inflammatory cytokines TNF‐α and IFN‐γ while suppressing the anti‐inflammatory cytokine IL‐10 (Figure 3E).

To further establish the contribution of ITGAM^+^ sEV to CD8^+^ T cell activation in vivo, an anti‐ITGAM neutralizing antibody (ITGAM‐nAb) was applied to counteract their function prior to IRI surgery. Consistently, ITGAM‐nAb pretreatment resulted in a substantial attenuation of renal injury (Figure S3A,B), with significantly reduced tubular injury scores and TUNEL^+^ apoptotic cells compared to controls. Immunofluorescence and flow cytometric analyses revealed markedly diminished CD8^+^ T cell infiltration into the injured kidney (Figure S3C,F), accompanied by a pronounced reduction in IFN‐γ‐producing CD8^+^ effector T cells (Figure S3G). Renal transcription of pro‐inflammatory cytokines IL‐1β and IL‐6 was also decreased upon ITGAM‐nAb treatment (Figure S3D,E). Collectively, these results establish that ITGAM^+^sEV represent a distinct subpopulation of renal immune microenvironment that drives CD8^+^T cell activation.

### 3.4 In Vivo Genetic Promotion of Endogenous ITGAM^+^ EV Secretion Drives CD8^+^ T Cell Activation

We next sought to determine the *in vivo* relevance of ITGAM^+^sEV with an CD63‐PAGFP^ITGAM‐KI^ reporter mouse model, which enables promoted EV secretion and their PAGFP labelling from ITGAM^+^ myeloid cells (Figure 4A). Following IRI, a marked upregulation of ITGAM and CD63 expression in the kidneys of CD63‐PAGFP^ITGAM‐KI^ mice was validated compared to sham controls (Figure 4B). Consistent with this, both fluorescent NTA and nanoparticle flow cytometry revealed a significantly higher abundance of PA‐GFP^+^ particles isolated from kidney (Figure 4C,D). Hence, a genetic mice model characterized with increased production of EVs from ITGAM^+^ myeloid cells with traceability was successfully established.

**FIGURE 4:**
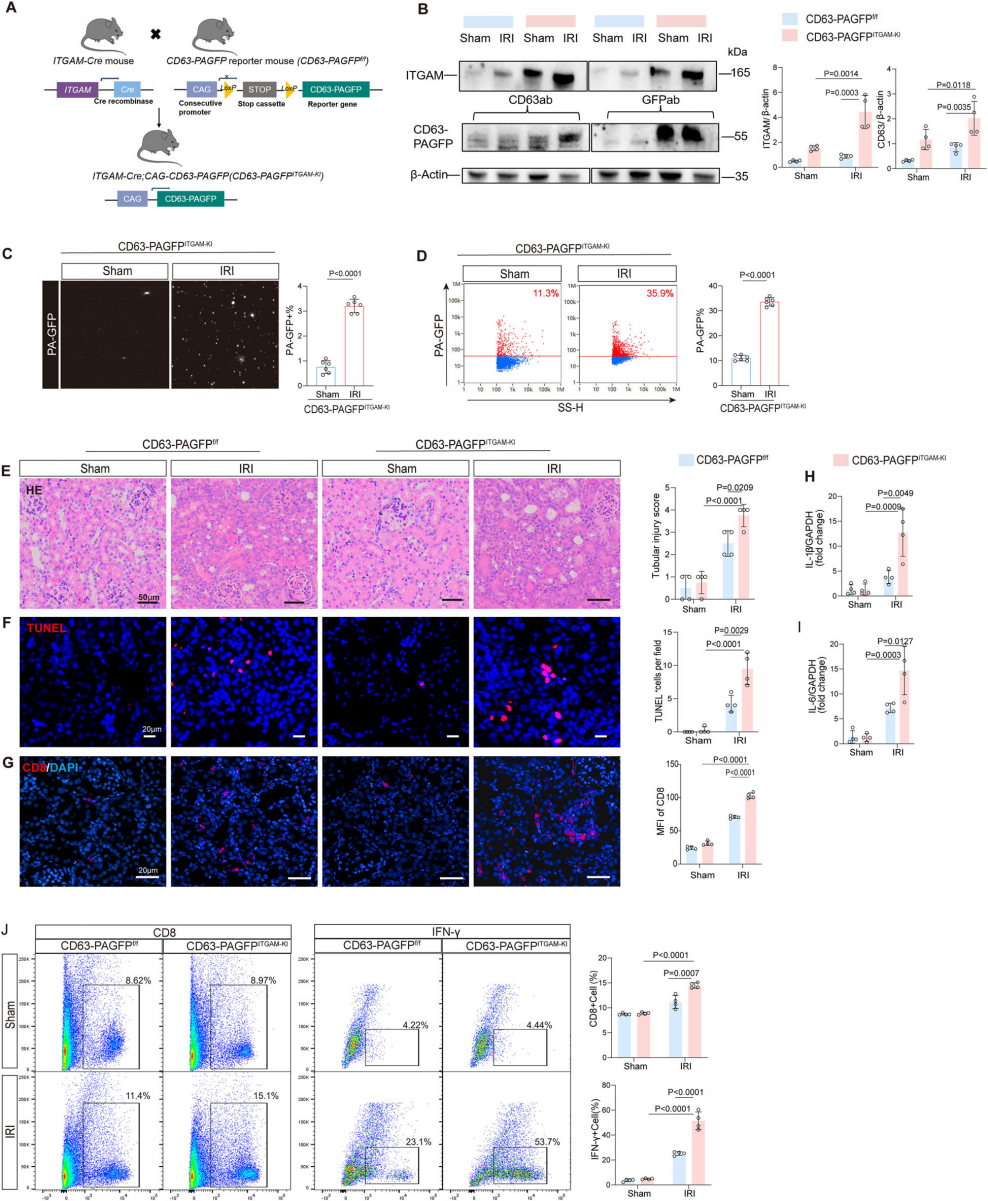
Generation of CD63‐PAGFP^ITGAM‐KI^ reporter mice enhances CD8^+^ T cell infiltration after IRI. (A) Schematic diagram for the generation of the CD63‐PAGFP^ITGAM‐KI^ reporter mice. (B) Western blot analysis of ITGAM and CD63 expression in kidney lysates from CD63‐PAGFP^f/f^ and CD63‐PAGFP^ITGAM‐KI^ mice (*n* = 4). (C) Fluorescent NTA and (D) nanoFCM analysis of the proportion of PAGFP^+^ sEV in the IRI group compared to the sham group (*n* = 6). (E–G) Analysis of renal injury at day 1 post‐IRI. (E) Representative Hematoxylin and Eosin (HE) staining, with quantification of tubular injury scores. Scale bars, 50 µm. (F) Representative images of TUNEL staining (red) and quantification of TUNEL^+^ cells. (G) Immunofluorescence staining of CD8^+^ T cells (red) in kidney sections and quantification of mean fluorescence intensity (MFI) (*n* = 4). Scale bars, 20 µm. (H) IL‐1β and (I) IL‐6 mRNA expression in CD63‐PAGFP^ITGAM‐KI^ or CD63‐PAGFP^f/f^ mice post‐IRI compared to control (*n* = 4). (J) Flow cytometric analysis of CD8^+^ T cells and IFN‐γ^+^CD8^+^ T cells in kidney tissue (*n* = 4). Statistical significance for (C, D) was determined by a two‐tailed unpaired Student's *t*‐test. For (B, E–J), one‐way ANOVA followed by Tukey's multiple comparisons test was used.

Impressively, CD63‐PAGFP^ITGAM‐KI^ mice subjected to IRI exhibited aggravated renal injury and inflammation at day1 after IRI‐AKI, Histologically, this manifested as significantly higher tubular injury scores (Figure 4E) and a profound elevation in cell apoptosis by TUNEL assay (Figure 4F). This exacerbated tissue damage was accompanied by a more robust inflammatory milieu, featuring a greater influx of CD8^+^ T cells and augmented renal transcription of pro‐inflammatory cytokines IL‐1β and IL‐6 (Figure 4G–I). Furthermore, flow cytometric analysis demonstrated a significant expansion in the proportion of CD8^+^T cells and an increase in IFN‐γ‐producing cells in the IRI group of CD63‐PAGFP^ITGAM‐KI^ mice (Figure 4J). Besides, using the bilateral IRI model, CD63‐PAGFP^ITGAM‐KI^ mice exhibited significantly elevated serum creatinine and blood urea nitrogen levels compared with CD63‐PAGFP^f/f^ controls, confirming exacerbated renal dysfunction, alongside aggravated renal injury, inflammatory cell infiltration, and pro‐inflammatory cytokine responses. (Figure S4). Therefore, *in vivo* data demonstrated that the expansion of ITGAM^+^sEV leads to enhanced activation of CD8^+^T cells after AKI.

### 3.5 Dynamic Visualization Reveals Active Interaction Between ITGAM^+^sEV and CD8^+^T Cell in Renal Lymphatic Microenvironment

To uncover the real‐time interplay of ITGAM^+^sEV and CD8^+^ T cells within the injured kidney, spatial and dynamic visualization of *in vivo* PAGFP labelled ITGAM^+^sEV was performed. Impressively, ITGAM^+^sEV^IRI^ (PA‐GFP^+^) and CD8^+^T cells (yellow, anti‐CD8) were markedly increased and frequently present in the area around renal lymphatic vessels (red, anti‐LYVE‐1) (Figure 5A).

**FIGURE 5:**
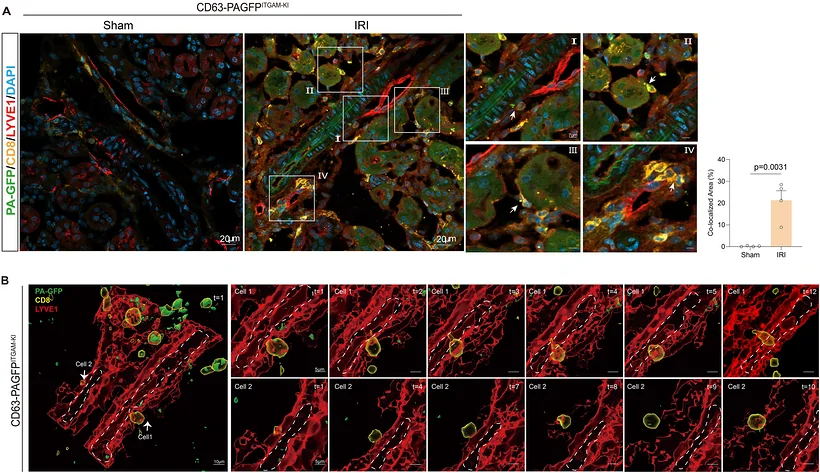
ITGAM^+^ sEV colocalize and dynamically interact with CD8^+^ T cells in the renal lymphatic vessel region in vivo. (A) Representative immunofluorescence images showing co‐staining of renal lymphatic vessels (LYVE1, red), CD8^+^ T cells (yellow), and ITGAM^+^ sEV (PA‐GFP, green) in kidneys from CD63‐PAGFP^ITGAM‐KI^ mice after sham or IRI surgery. Quantification of PA‐GFP signal within the CD8^+^ T cell area is shown on the right (unpaired two‐tailed unpaired t test, *n* = 4. Data are presented as mean ± SD.). (B) Representative time‐lapse confocal images illustrating dynamic cellular interactions, as detailed in Movie S1.

To capture the spatial and temporal dynamics of this interaction, live kidney‐slice was cultured and imaged over a period of time. Interestingly, two distinct behaviours of CD8^+^T cells interacting with PAGFP labelled sEV were noticed in kidney of IRI mice, i.e., CD8^+^T cells bearing PAGFP^+^sEV migrated from the perilymphatic interstitium into the lymphatic lumen, or CD8^+^T cells exited the lymphatic vessel to seek and internalize PAGFP^+^sEV (Figure 5B, Movie S1). In contrast, no detectable PAGFP^+^sEV was noticed in live imaging of sham‐operated controls, meanwhile, transmigration was not observed for CD8^+^T cells within the lymphatic vessels (Movie S2). These observations demonstrate that the renal lymphatic maybe an important niche where ITGAM^+^ myeloid cell derived sEV actively regulate the trafficking and programming of CD8^+^T cells after renal injury.

### 3.6 Surface S100a9 as Function Cargo of ITGAM^+^sEV Responsible for CD8^+^T Cell Activation During IRI‐AKI

To identify the molecular cargo contribute to the immunomodulatory activity of ITGAM^+^sEV, proteomic profiling on magnetically sorted ITGAM^+^sEV from IRI and Sham kidneys were analysed (Figure 6A). It was revealed that S100a9 was the most significantly upregulated protein in ITGAM^+^sEV from IRI kidneys compared to sham controls, hierarchical clustering analysis also revealed S100a9 as the top‐ranking differentially expressed protein (Figure 6B,C). Immunoelectron microscopy validated that S100a9 prominently displayed on the EV surface (Figure 6D). dSTORM super‐resolution imaging visualized S100a9 molecules on single ITGAM^+^sEV (Figure 6E). To confirm the location of S100a9 on ITGAM^+^ sEV^IRI^, nano‐flow cytometry was performed using antibodies against S100a9 and the integral EV membrane protein CD63 on intact or TritonX‐100‐permeabilized vesicles. The proportion of S100a9^+^CD63^+^ particles were comparable between intact and permeabilized conditions (4.51% vs. 4.66%, p = ns, Figure 6F), indicating that S100a9 was predominantly located on the EV surface. Importantly, quantitative analyses using nanoparticle flow cytometry and Western blotting demonstrated an elevated S100a9 expression in ITGAM^+^sEV from IRI kidneys compared to sham controls (Figure 6G,H). Next, the contribution of surface S100a9 to CD8^+^T cells activation was investigated. It was identified that enzymatic digestion of surface proteins markedly reduced IFN‑γ production in CD8^+^T cell induced by ITGAM^+^sEV^IRI^ (Figure 6I). More specifically, pharmacological inhibition of S100a9 using tasquinimod (TASQ) similarly attenuated the upregulation of IFN‐γ and TNF‐α production induced by ITGAM^+^sEV, while increased the level of regulatory cytokine IL‐10 (Figure 6J–L). Impressively, we found that ITGAM^+^sEV^IRI^ were more potent at inducing IFN‐γ in CD8^+^ T cells than soluble recombinant S100a9(solS100a9) at equal molecule concentration, demonstrating that the EV‐associated context is required for the maximal pro‐inflammatory activity of S100a9 (Figure 6M). Genetic loss‐of‐function experiments were further performed using S100a9‐knockout (S100a9‐KO) mice. ITGAM^+^ myeloid cells were isolated from S100a9‐KO and WT littermates, and their derived EVs (ITGAM^+^sEV^IRI(WT)^ and ITGAM^+^sEV^IRI(S100a9‐KO)^) were purified (Figure S5A–D). Compared with ITGAM^+^sEV^IRI(WT)^, ITGAM^+^sEV^IRI(S100a9‐KO)^ markedly attenuated CD8^+^ T cell activation, as evidenced by reduced Ki‐67^+^ and IFN‐γ^+^ populations, lower TNF‐α secretion, and elevated IL‐10 production (Figure S5E–I).

**FIGURE 6:**
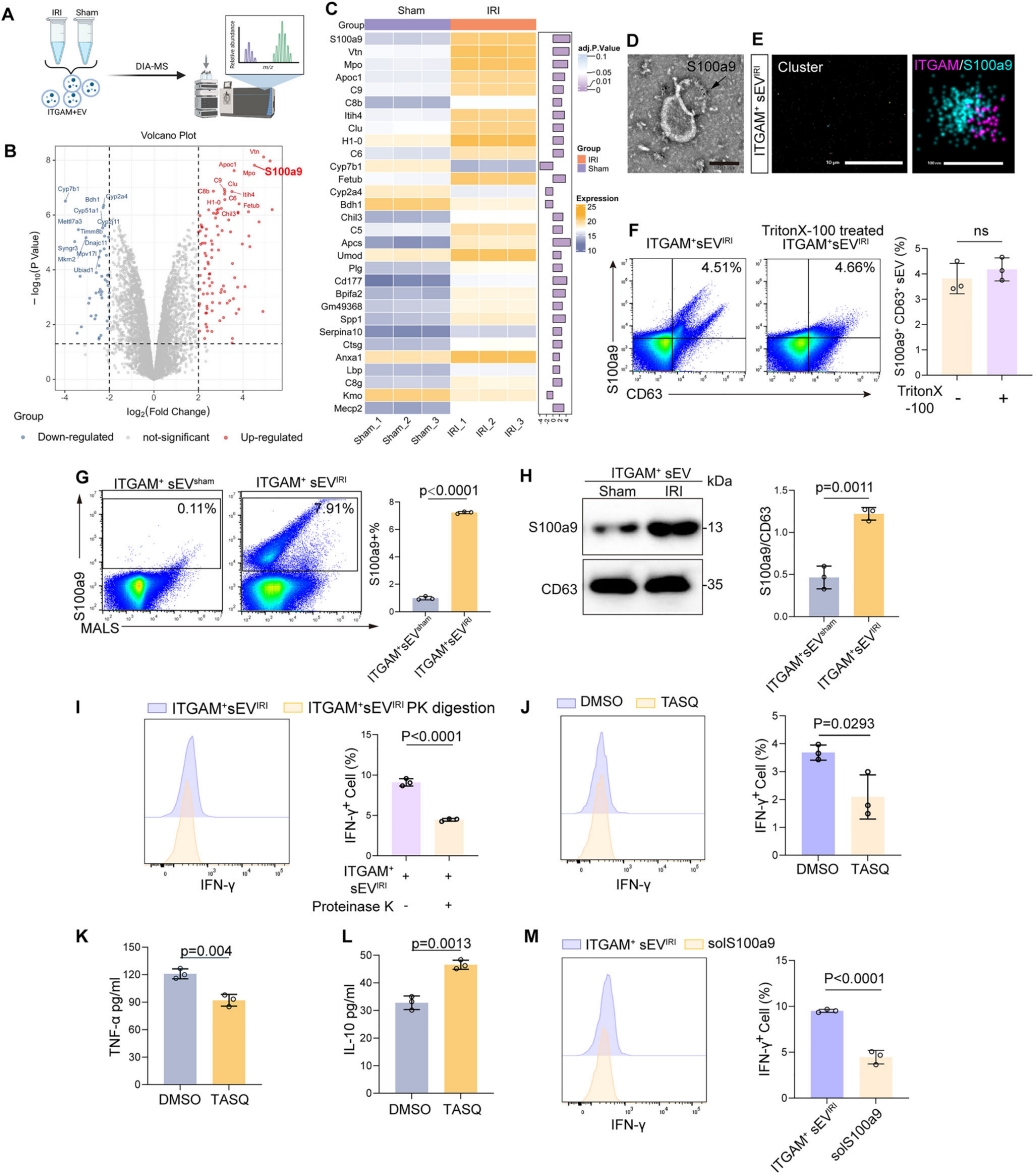
S100a9 is identified as a key protein in ITGAM^+^ EV that modulates CD8^+^ T cell proliferation and activation in vitro. (A) Schematic overview of proteomic profiling of ITGAM^+^ sEV. (B) Volcano plot showing differentially expressed proteins in ITGAM^+^ sEV from IRI versus sham mice. (C) Heatmap of differentially expressed proteins in ITGAM^+^ EVs from sham and IRI groups. The rightmost column indicates the adjusted *p*‐value (*n* = 3). (D) Representative immunoelectron microscopy image of ITGAM^+^ sEV from IRI kidney, with the arrow indicating S100a9 localization. Scale bar, 100 nm. (E) Representative dSTORM super‐resolution images showing ITGAM (purple) and S100a9 (blue) on single sEV from the IRI group. Scale bars, 10 µm (clusters) and 100 nm (magnified view). (F) nano‐flow cytometry analysis of the surface localization of S100a9 on ITGAM^+^ sEV^IRI^. EVs were co‐immunostained with anti‐S100a9 and anti‐CD63 either without (−TritonX‐100) or following membrane permeabilization with buffer (+TritonX‐100). Quantification of the percentage of S100a9^+^CD63^+^ sEV is shown on the right (*n* = 3). ns, not significant. (G) nanoFCM and (H) Western blot analysis of S100a9 expression in ITGAM^+^ sEV from sham and IRI group (*n* = 3). (I) Flow cytometric quantification of IFN‐γ^+^CD8^+^ T cells after co‐culture with ITGAM^+^ sEV from IRI mice that were either untreated or pre‐treated with proteinase K (PK) (*n* = 3). (J) Quantification of IFN‐γ^+^CD8^+^ T cells by flow cytometry, and TNF‐α (K) and IL‐10 (L) levels in the culture supernatant after co‐culture of CD8^+^ T cells with ITGAM^+^ sEV^IRI^ in the presence of the S100a9 inhibitor Tasquinimod (TASQ) or vehicle control (DMSO) (*n* = 3).(M) Quantification of IFN‐γ^+^CD8^+^ T cells comparing the effects of vesicular versus soluble S100a9. CD8^+^ T cells were treated with either ITGAM^+^ EVs from IRI mice (ITGAM^+^sEV^IRI^) or an equivalent concentration of soluble recombinant S100a9 (solS100a9). Data are presented as mean ± SD. For (F–M), statistical significance between two groups was determined using a two‐tailed unpaired Student's *t*‐test.

*In vivo*, CD63‐PAGFP^ITGAM‐KI^ reporter mice were treated with the S100a9 inhibitor tasquinimod prior to undergoing IRI. In this genetic model, which was characterized by increased EV secretion and aggravated inflammation post‐IRI as we have already established, tasquinimod treatment markedly attenuated interstitial inflammation at day 1 when compared to DMSO‐treated controls. This improvement was reflected by reductions in tubular damage on H&E staining, a noticeable decline in apoptotic cells, diminished infiltration CD8^+^ T‐cell and a downregulation of pro‐inflammatory cytokines IL‐1β and IL‐6, indicating that S100a9 was the principle functional cargo that promoted the early activation of T cells after AKI (Figure 7A–F). Flow cytometry confirmed reduced renal CD8^+^T cells and diminished IFN‐γ production induced by tasquinimod treatment (Figure 7G,H).

**FIGURE 7:**
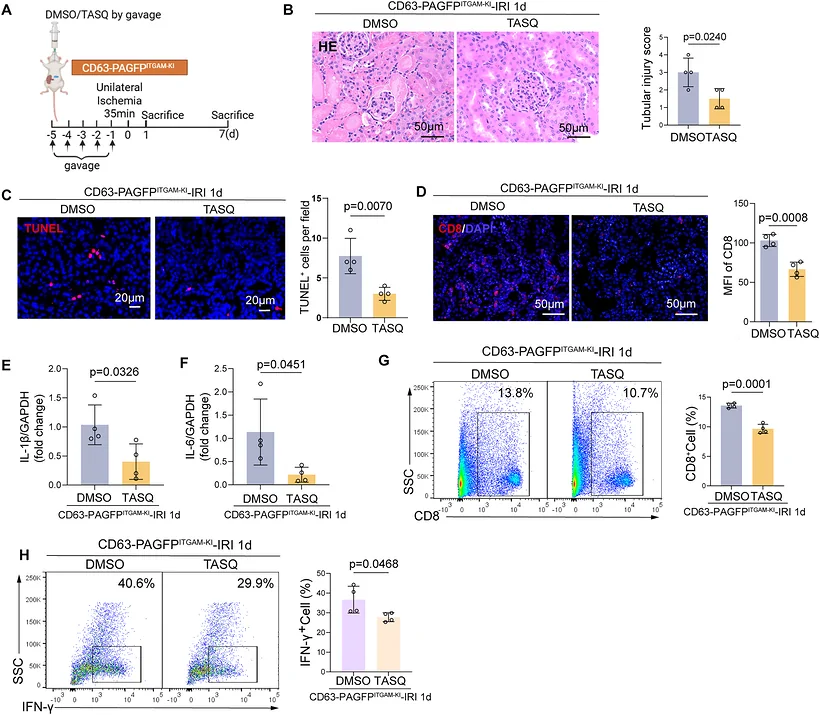
Pharmacological inhibition of S100a9 ameliorates CD8^+^ T Cell Infiltration and renal inflammation in vivo. (A) CD63‐PAGFP^ITGAM‐KI^ mice were subjected to uIRI and treated with either the S100a9 inhibitor TASQ or vehicle control (DMSO) via oral gavage, as depicted in the schematic diagram. (B) Representative H&E staining of kidney sections from DMSO‐ and tasquinimod‐treated mice. Scale bars, 50 µm; right, quantification of tubular injury score (*n* = 4). (C) Representative images and quantification of TUNEL staining (red) (*n* = 4). (D) Representative immunofluorescence staining of CD8 (Red) in kidney sections. Scale bars, 50 µm; right, quantification of CD8 mean fluorescence intensity (MFI) (*n* = 4). (E) IL‐1β and (F) IL‐6 mRNA expression in two groups (*n* = 4). (G, H) Flow‐cytometric analysis of renal CD8^+^ T cells (G) and IFN‐γ‐producing cells among the CD8^+^ T cell population (H) in the kidneys from DMSO‐ and tasquinimod‐treated mice (*n* = 4). For B–H, statistical significance between the two treatment groups (DMSO vs. TASQ) was determined using a two‐tailed unpaired Student's *t*‐test. Data are presented as mean ± SD.

Given that T cell infiltration continues to expand during the later, adaptive immune phase of AKI, we next sought to determine if S100a9 inhibition also impacts this sustained response. Indeed, analysis at day 7 post‐IRI revealed that tasquinimod treatment continued to substantially mitigate the accumulation of CD8^+^ T cells, demonstrating the role for this pathway in T cell recruitment and/or retention (Figure S6A–G).

To test the generalizability of this mechanism beyond ischemic injury, we administered the S100a9 inhibitor tasquinimod in a model of cisplatin‐induced nephrotoxicity (Figure S7A). Similar to the IRI setting, tasquinimod treatment markedly reduced renal inflammatory responses, evidenced by lower IL‑1β and IL‑6 expression, diminished histologic injury on HE staining, decreased apoptotic cells on TUNEL staining, reduced CD8^+^ T‑cell accumulation by immunofluorescence, and lower frequencies of renal CD8^+^ T cells and their IFN‑γ^+^ subsets by flow cytometry (Figure S7B–H). Together, these findings indicate that surface‑expressed S100a9 on ITGAM^+^sEV^IRI^ is the key component to enhance CD8^+^ T‑cell effector function across different AKI etiologies.

### 3.7 ITGAM^+^ EV‐delivered S100a9 Interacts With Mitochondrial Slc25a12 in CD8^+^ T Cells

To define the underlying mechanism, proteomics analysis on CD8^+^T cells treated with ITGAM^+^sEV^IRI^, ITGAM^+^sEV^IRI^ in the presence of tasquinimod, or vehicle control was performed. Notably, ITGAM^+^sEV^IRI^ treatment resulted in upregulated proteins predominantly enriched in mitochondrial metabolic processes, including ATP synthesis, the tricarboxylic acid (TCA) cycle, and respiratory chain complex assembly. which was absent in untreated controls and abrogated by S100a9 inhibition with tasquinimod (Figure 8A–C). Motivated by these findings, we performed immunoprecipitation–mass spectrometry (IP–MS) using an anti‐S100a9 antibody on lysates from ITGAM^+^sEV^IRI^ ‐treated CD8^+^T cells. Among the top candidates, Slc25a12, the mitochondrial aspartate/glutamate carrier located on the inner mitochondrial membrane was identified (Figure 8D).

**FIGURE 8:**
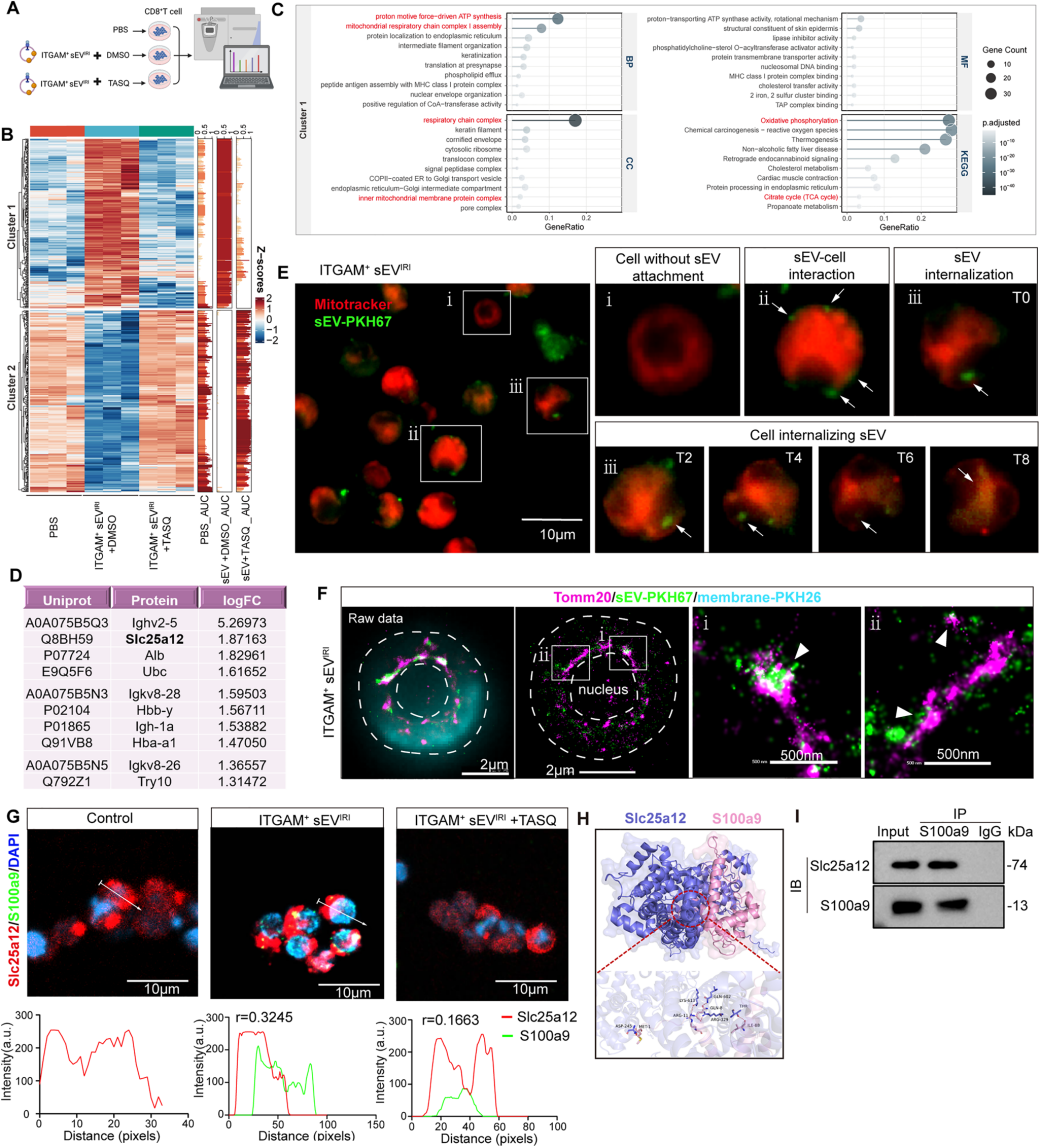
ITGAM^+^ EV–delivered S100a9 targets mitochondrial Slc25a12 in CD8^+^ T cells. (A) Schematic diagram of the experimental design for proteomic analysis of CD8^+^ T cells treated with PBS (control), ITGAM^+^ sEV^IRI^ plus solvent (DMSO) or ITGAM^+^ sEV^IRI^ plus the S100a9 inhibitor TASQ. (B) Hierarchical clustering heatmap of differentially expressed proteins. (C) Integrated pathway enrichment analysis (Gene Ontology and KEGG) of proteins within Cluster 1. (D) Top‐ranking candidate proteins identified by immunoprecipitation coupled with mass spectrometry (IP‐MS) using an anti‐S100a9 antibody on lysates from ITGAM^+^ sEV^IRI^‐treated CD8^+^ T cells. (E) Representative time‐lapse live‐cell imaging capturing the sequential process of ITGAM^+^ sEV^IRI^ (green, PKH67‐labeled) attachment, interaction, and internalization by a CD8^+^ T cell, followed by subsequent localization to mitochondria (red, MitoTracker). (F) dSTORM super‐resolution imaging demonstrating the colocalization of an internalized ITGAM^+^ sEV^IRI^ (green) with the mitochondrial outer membrane marker TOMM20 (purple) within a CD8^+^ T cell. The cell membrane is stained red (pseudocolored blue) for clarity. Scale bar, 500 nm. (G) Representative immunofluorescence images showing colocalization of S100a9 (green) and Slc25a12 (red) in CD8^+^ T cells following treatment with ITGAM^+^sEV^IRI^. This colocalization is absent in untreated controls and markedly diminished by co‐treatment with TASQ. Pearson's correlation coefficients (r) are provided in the images. Scale bars, 10 µm. (H) Molecular docking model of the S100a9‐Slc25a12 complex, showing the predicted interaction interface (left) and key contact residues (magnified view, bottom). (I) Co‐immunoprecipitation (Co‐IP) using an anti‐S100a9 antibody or IgG control on lysates from ITGAM^+^sEV^IRI^‐treated CD8^+^ T cells, followed by immunoblotting for Slc25a12.

To visualize the delivery of S100a9 via ITGAM^+^sEV^IRI^ to CD8^+^T cells, live‐cell imaging of CD8^+^T cells was treated with PKH26‐labeled ITGAM^+^sEV and stained with mitotracker meanwhile. Impressively, we observed a continuing process of EV approaching to T cells, adhesion to cellular surface followed by internalization which subsequently localized to mitochondria (Figure 8E, Movie S3). In contrast, ITGAM^−^sEV^IRI^ were not taken up by CD8^+^ T cells over the same time course observed, underscoring the requirement of ITGAM‐dependent internalization (Figure S8). Super‐resolution dSTORM imaging demonstrated clear colocalization of internalized EVs with mitochondria (Figure 8F). Impressively, confocal imaging revealed robust colocalization between S100a9 and Slc25a12 following ITGAM^+^sEV treatment which was diminished remarkably upon tasquinimod treatment (Figure 8G). To obtain direct evidence for a physical interaction between S100a9 and Slc25a12, molecular docking analysis was performed, predicting a stable protein complex characterized by a large interaction interface and a favourable binding free energy, with defined contact residues at the interface (Figure 8H). This predicted interaction was further validated by co‐immunoprecipitation experiment (Figure 8I). Together, these data indicate that S100a9 delivered by ITGAM^+^sEV reaches the mitochondrial in CD8^+^T cells and associates spatially with Slc25a12 which may contribute to the activation of CD8^+^T cells.

### 3.8 S100a9 Promotes Mitochondrial Amino Acid Transport by Stabilizing Slc25a12 in CD8^+^T Cells

We proceeded to investigate the functional relevance of the S100a9‐Slc25a12 interaction and CD8^+^T cell activation. *In vitro*, tasquinimod treatment reduced Slc25a12 protein expression in CD8^+^T cells induced by ITGAM^+^sEV^IRI^, indicating S100a9 is required to maintain Slc25a12 expression (Figure 9A,B). We further found that S100a9 inhibition via tasquinimod significantly impaired mitochondrial function of CD8^+^T cells, as evidenced by decreased ATP production and a lower NAD^+^/NADH ratio compared to treatment with ITGAM^+^sEV^IRI^ alone (Figure 9C,D). As the primary mitochondrial aspartate‐glutamate carrier, Slc25a12 is directly responsible for transporting glutamate into the mitochondrial matrix to fuel the TCA cycle and support T‐cell bioenergetics. Accordingly, we assessed both cytosolic and mitochondrial glutamate levels and found that S100a9 inhibition reduced mitochondrial glutamate while causing a concomitant increase in cytosolic glutamate, consistent with impaired Slc25a12‐mediated transport (Figure 9E,F). To directly test the requirement of Slc25a12 for fuelling mitochondrial metabolism, siRNA‐mediated knockdown of Slc25a12 significantly blunted the metabolism induced by ITGAM^+^sEV^IRI^ (Figure 9G–J).

**FIGURE 9:**
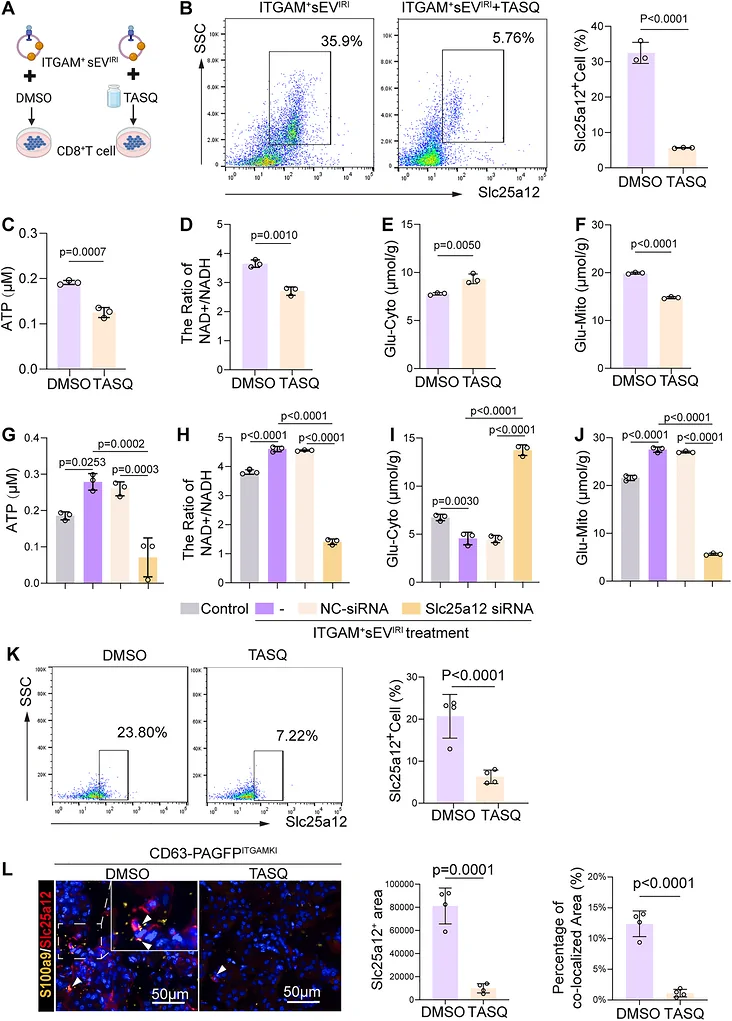
Inhibition of ITGAM^+^ EV–mediated S100a9 suppresses Slc25a12 expression and mitochondrial metabolism in CD8^+^ T cells. (A) Schematic of the experimental setup: CD8^+^ T cells were treated with ITGAM^+^ sEV^IRI^ in the presence of either the S100a9 inhibitor TASQ or DMSO. (B) Flow cytometric analysis of Slc25a12 expression in CD8^+^ T cells of TASQ and DMSO groups (*n* = 3). (C–F) Detection of mitochondrial metabolic indicators of CD8^+^ T cells in TASQ and DMSO groups, including (C) cellular ATP content, (D) NAD+/NADH ratio, (E) intracellular glutamate content, and (F) mitochondrial glutamate content (*n* = 3). (G) ATP levels, (H) NAD^+^/NADH ratio, (I) intracellular glutamate, and (J) mitochondrial glutamate were measured in four groups: untreated CD8^+^ T cells (control), CD8^+^ T cells treated with ITGAM^+^ sEV^IRI^, CD8^+^ T cells transfected with a non‐targeting control siRNA (siNC) prior to ITGAM^+^ sEV^IRI^, and CD8^+^ T cells transfected with siRNA targeting Slc25a12 prior to ITGAM^+^ sEV^IRI^ (*n* = 3). K) CD63‐PAGFP^ITGAM/KI^ mice were orally administered TASQ or DMSO after IRI, and renal tissue single‐cell suspensions were subjected to flow cytometry to analyse Slc25a12 expression in CD8^+^ T cells (*n* = 4). (L) Immunofluorescence colocalization analysis of renal tissue sections, showing the colocalization of S100a9 with Slc25a12 in both groups (DMSO vs. TASQ), and quantification of the colocalization area (*n* = 4). Data are presented as mean ± SEM. Statistical significance between two groups (B–F, K, L) was determined using a two‐tailed unpaired Student's *t*‐test. For multiple group comparisons (G–J), a one‐way ANOVA followed by Tukey's post‐hoc test was used.

*In vivo*, using the same 1‐day post‐IRI model shown in Figure 7A, oral administration of tasquinimod diminished the colocalization of S100a9 and mitochondrial Slc25a12 in renal tissues. Besides, flow cytometric analysis of single‐cell suspensions from kidney tissue identified decreased Slc25a12 expression in CD8^+^T cells, confirming that S100a9 activity is essential for maintaining Slc25a12 expression *in vivo*. (Figure 9K,L). Collectively, these data demonstrate that S100a9 potentially sustains Slc25a12 protein stability, thereby preserving mitochondrial amino acid transport capacity and sustaining the enhanced metabolic state required for effector T‐cell function.

## 4 Discussion

In this study, surface proteome of single EV was profiled to delineate the EV‐associated immune microenvironment in IRI induced AKI, ITGAM^+^sEV derived from myeloid cells was identified as predominant subpopulation with remarkable changes which drives CD8^+^T cell activation. Mechanistically, surface‐displayed S100a9 on ITGAM^+^sEV derived from injured kidney promotes CD8^+^T cell mitochondrial amino acid transport by stabilizing the carrier Slc25a12. This effects consequently promoted mitochondrial metabolism, which in turn enhanced the pro‐inflammatory function of CD8^+^T cells. These findings establish ITGAM^+^sEV‐delivered S100a9 that stabilized Slc25a12 as a key initiator of pathogenic CD8^+^ T cell activation in AKI, underscoring its potential as a therapeutic target via blocking the communication of innate and adaptive immunity.

The surface proteome profile at single‐vesicle resolution identified a shift of EV composition toward immune cell‐derived EVs in kidney of IRI‐AKI. This mapping of EV subpopulation identifies that EVs from myeloid cells, that is macrophages, dendritic cells, and neutrophils, constitute the predominant subpopulations of the EVs with remarkable proportional changes at day 1 after AKI. While immune cell‐derived EVs have been widely studied on their functional roles in immune regulation, whether they form distinct, local tissue immune microenvironment has remained largely unexplored (Niel and G.D'Angelo 2018, Kalluri and LeBleu 2020). Our work provides the single‐EV resolved map of the EV‐associated immune landscape within the injured kidney during AKI. This finding provided a new insight into AKI immune microenvironment constituent of EVs beyond infiltrating cells and soluble mediators (Lee et al. 2024, Jang and Rabb 2015).

Further, we identified a subset of ITGAM^+^sEV derived from myeloid cells, as a crucial driver of pathological CD8^+^T cell activation in AKI. This conclusion is substantiated by functional assays using immuno‐purified ITGAM^+^sEV subsets and *in vivo* validation with a CD63‐PAGFP^ITGAM‐KI^ reporter mouse model, which demonstrated that genetically enhancing the secretion of these specific EVs aggravates CD8^+^T cell‐mediated inflammation. Critically, we also captured the dynamic interactions between ITGAM^+^sEV and CD8^+^T cells in real‐time within the injured kidney, revealing a spatial interplay near the lymphatic vessels. This vesicle‐dependent mechanism for innate‐adaptive immune crosstalk represents a distinct mode of signalling. Unlike soluble factors like cytokines, which are prone to dilution and degradation in the extracellular space, EVs act as stable vectors that protect their cargo, enabling the delivery of a concentrated signal over greater distances within a tissue (Poggio et al. 2019). Moreover, this process circumvents the requirement for direct, sustained physical contact necessary for a classical immunological synapse (Dustin and Long 2010). Herein, we demonstrate that a defined myeloid‐derived ITGAM^+^sEV subpopulation serve as a key driver of the CD8^+^T cell activation at the early stage after AKI which may represent the priming signal that initiates the adaptive immune response sent from myeloid cells (Iwasaki and Medzhitov 2015). However, advances in imaging technology are still needed to trace the interaction more clearly between endogenous EVs and their target cells *in vivo*. Besides, further studies are needed to determine whether ITGAM^+^ EVs preferentially interact with CD8^+^ T cells over other immune cell populations.

To investigate the interplay of ITGAM^+^sEV with CD8^+^T cell *in vivo* (He et al. 2024, Zhu et al. 2023), real‐time visualization the interactions within the kidney was realized through a genetically engineered CD63‐PAGFP^ITGAM‐KI^ reporter system. We identified their interaction are spatially concentrated in the renal perilymphatic interstitium, where CD8^+^T cells exhibit distinct trafficking behaviours. On one hand, T cells internalize ITGAM^+^ EVs and enter lymphatic lumens, a behaviour consistent with evidence demonstrating that inflammatory signals license T cells to sense egress gradients for systemic trafficking (Baeyens et al. 2021). On the other hand, we observed T cell exiting from lymphatics back into the interstitium, implying a localized retention signal. We propose that the accumulation of ITGAM^+^sEVs in the perilymphatic area establishes a retentive niche that, analogous to S1P‐mediated retention signals (Campos et al. 2025), draws T cells back from lymphatic vessels to reinforce their local effector function. Together, these distinct trafficking behaviours suggest that ITGAM^+^sEV may influence both the behaviours of CD8^+^T cells, although further investigation is required to determine the extent and mechanism of their involvement.

Mechanistically, we demonstrate that ITGAM^+^sEV deliver S100a9 into CD8^+^T cells, where it acts on the inner mitochondrial membrane carrier Slc25a12. This interaction enhances mitochondrial amino acid transport, promoting CD8^+^T cell metabolic capacity and pro‐inflammatory capacity. As a canonical DAMP, S100a9 is an extracellular signal that drives inflammation via surface receptors like TLR4 and RAGE (Wang et al. 2018), which is also a definitive marker for inflammatory macrophage subsets (Yao et al. 2022). Interestingly, recent research has begun to uncover its non‐canonical intracellular function. For instance, the translocation of lactylated S100a9 into the neutrophil nucleus function as a transcriptional co‐activator (Wang et al. 2025). Our work showed that S100a9 can be trafficked via EVs to a distinct cellular compartment, that is the mitochondria in CD8^+^T cells. Slc25a12, the mitochondrial aspartate‐glutamate carrier, is significant for T‐cell bioenergetics: it supports cellular respiration by facilitating the malate‐aspartate shuttle for redox balance and fuels protein biosynthesis by providing glutamate precursors essential for clonal expansion and differentiation of activated T cells (Norton et al. 2025, Liu and Peng 2021, Monné et al. 2019). By demonstrating that EV‐delivered S100a9 promoted the stability of the core metabolic gatekeeper Slc25a12, we define the ITGAM^+^sEV–S100a9–Slc25a12 axis that links myeloid cells to CD8^+^T cell mitochondrial metabolism. This also illustrates that a canonical DAMP, when packaged in EVs, can function intracellularly to reprogram mitochondrial metabolism in T cells which is distinct from classical extracellular receptor–ligand signalling.

The identification of the ITGAM^+^sEV‐S100a9 in transferring signals from innate immunity to adaptive CD8^+^T cell activation reveals promising therapeutic opportunities. Correspondingly, pharmacologic inhibition of S100a9 with tasquinimod, attenuated CD8^+^ T cell responses in both ischemia‐reperfusion and cisplatin‐induced renal injury, whereas genetic ablation of S100a9 restricted to bone marrow‐derived myeloid cells yielded comparable protective effects specifically in the IRI model. This extends the therapeutic rationale for S100a9 inhibitors beyond the canonical disruption of extracellular S100a9‐receptor interactions (Björk et al. 2009). Given that tasquinimod is a cell‐permeable small molecule (Isaacs et al. 2012), we therefore propose that it can neutralize DAMP signalling and disrupt the intracellular function of S100a9 delivered by EV within T cells. Collectively, these findings suggest that targeting critical cargo of ITGAM^+^sEV offers a promising strategy to restrain continued inflammation resulting from adaptive immune response in AKI.

In conclusion, by integrating single‐vesicle proteome analysis with functional validation, our study establishes ITGAM^+^ sEV sourced from myeloid cells as critical mediators that initiate the early activation of pathogenic T cells in AKI through S100a9‐Slc25a12 mediated mitochondria metabolism. The delineation of this axis provides critical insight into AKI pathogenesis and establishes a target for therapeutic intervention against kidney injury.

## Author Contributions

**Yu‐Qi Fu**: writing – original draft, investigation, formal analysis, conceptualization, visualization. **Yi‐Lin Zhang**: supervision, methodology, conceptualization, investigation, data curation. **An‐Ran Shen**: methodology, writing – original draft, formal analysis, investigation, data curation. **Ning Li**: methodology, data curation, formal analysis, software. **Jing Song**: methodology, data curation, formal analysis, validation. **Yan Bao**: methodology, data curation, formal analysis, visualization. **Si‐Meng Wang**: methodology, data curation, formal analysis. **Ding‐Ding Xue**: methodology, data curation, formal analysis. **Min Wu**: methodology, data curation, formal analysis. **Bin Wang**: methodology, data curation, formal analysis. **Yi Wen**: methodology, data curation, formal analysis. **Tao‐Tao Tang**: supervision, conceptualization, validation, data curation. **Lin‐Li Lv**: writing – review and editing, project administration, supervision. **Bi‐Cheng Liu**: resources, supervision, project administration, funding acquisition, writing – review and editing.

## Conflicts of Interest

The authors declare no conflicts of interests.

## Supporting information




**Supporting Information**: jev270404‐sup‐0001‐FigureS1‐S8.docx

**Supporting Information**: jev270404‐sup‐0002‐TableS1‐S2.docx

**Supporting Information**: jev270404‐sup‐0003‐SuppMat.docx

**Supporting Information**: jev270404‐sup‐0004‐MovieS1.mp4

**Supporting Information**: jev270404‐sup‐0005‐MovieS2.mp4

**Supporting Information**: jev270404‐sup‐0006‐MovieS3.mp4

## Data Availability

All data supporting the findings of this study are available within the manuscript and supplementary materials. Additional datasets, including raw proteomics files, imaging data, and flow cytometry source files, are available from the corresponding authors upon reasonable request.
