# Supplementary material for: ITGAM+ Extracellular Vesicles Trigger CD8+ T Cell Activation via S100a9‐Mediated Metabolic Reprogramming in Acute Kidney Injury
Source: J Extracell Vesicles. 2026 Nov 5;15(11):e70404. doi: 10.1002/jev2.70404 (PMC13739760; doi:10.1002/jev2.70404)
Supplement: Supplementary file 1 [file JEV2-15-e70404-s001.docx]

**Supplementary Figures**

**Supplementary Figure 1. Quality control and EV/protein profiling across experimental groups.**

(A) Correlation matrix of renal EV proteomic profiles from individual sham and IRI mice (n=4). (B) Quantification of PBA‑detected total EV counts (left), total protein barcode counts (middle), and the protein‑per‑EV ratio (right) across sham and IRI samples (n=4).

**Supplementary Figure 2. Myeloid cells are the primary source of *Itgam* transcript in the IRI kidney.**

(A) Uniform manifold approximation and projection (UMAP) plot showing immune cell clusters annotated as neutrophils, macrophages, and dendritic cells derived from the public single‑cell dataset GSE267242. (B) Feature plot demonstrating that *Itgam* transcript expression is specifically localized to these three myeloid cell clusters.

**Supplementary Figure 3. In vivo neutralization of ITGAM attenuates CD8⁺ T cell activation and renal injury following IRI.** Mice were pretreated with anti-ITGAM neutralizing antibody (ITGAM-nAb, 500 μg/mouse, i.v.) or matched isotype IgG control 3 hours prior to unilateral IRI. Kidneys were harvested 24 hours post-reperfusion for analysis. (A) Representative H&E staining of kidney sections and quantification of tubular injury scores. Scale bars, 50 μm (n=4). (B) Representative TUNEL staining (red) and quantification of TUNEL⁺ cells per field. Scale bars, 50 μm (n=4). (C) Representative immunofluorescence staining of CD8 (red) with DAPI (blue) counterstain and quantification of CD8 mean fluorescence intensity (MFI). Scale bars, 50 μm (n=4). (D-E) IL-1β (D) and IL-6 (E) mRNA expression in kidney tissues quantified by RT-qPCR (n=4). (F) Representative flow cytometric analysis and quantification of CD8⁺ T cells in kidney single-cell suspensions (n=4). (G) Representative flow cytometric analysis and quantification of IFN-γ⁺ cells among CD8⁺ T cells (n =4). Data are presented as mean ± SD. Statistical significance was determined using a two-tailed unpaired Student's t-test.

**Supplementary Figure 4. Bilateral IRI(bIRI) CD63-PAGFP^ITGAM-KI^ reporter mice showed exacerbated renal injury.** Analysis of renal injury at day 1 post-bIRI. (A) Representative Hematoxylin and Eosin (HE) staining, with quantification of tubular injury scores. Scale bars, 50 μm. (B) Representative images of TUNEL staining (red) and quantification of TUNEL^+^ cells. (C) Immunofluorescence staining of CD8⁺ T cells (red) in kidney sections and quantification of mean fluorescence intensity (MFI) (n=4). Scale bars, 50 μm. (D) BUN, (E) Scr, (F) IL-1β, and (G) IL-6 mRNA expression in CD63-PAGFP^ITGAM-KI^ or CD63-PAGFP^f/f^ mice post-bIRI compared to control (n=4). Statistical significance was determined by one-way ANOVA, followed by Tukey's multiple comparisons test.

**Supplementary Figure 5. Genetic ablation of S100a9 in myeloid cell-derived EVs abolishes their pro-inflammatory activity on CD8⁺ T cells.** (A) Schematic illustrating the experimental workflow: ITGAM⁺ myeloid cells were isolated from the bone marrow and spleen of IRI-subjected WT and S100a9-KO mice via magnetic-activated cell sorting (MACS), followed by ultracentrifugation-based purification of ITGAM⁺sEV^IRI(WT)^ and ITGAM⁺sEV^IRI(S100a9-KO)^ for subsequent co-culture with CD8⁺ T cells. (B) Western blot analysis of S100a9 expression in kidney tissues from S100a9-KO and WT mice at day 1 post-IRI, demonstrating efficient knockout (P<0.0001, n=4). (C) Representative flow cytometric analysis of ITGAM⁺ cell purity before (Pre-sort) and after (Post-sort) magnetic-activated cell sorting. (D) Representative nano-flow cytometry plots and quantification of S100a9⁺ EVs among ITGAM⁺ sEV purified from WT and S100a9-KO myeloid cell cultures (35.2% vs 0.2%, P<0.0001, n=3). (E-F) Representative flow cytometry graphs and quantification of Ki-67⁺ (E), IFN-γ⁺ (F) CD8⁺ T cells following treatment with ITGAM⁺sEV^IRI(WT)^ or ITGAM⁺sEV^IRI(S100a9-KO)^ (n=3). (G,H) ELISA quantification of TNF-α (G) and IL-10 (H) levels in culture supernatants (n=3). Data are presented as mean ± SD. Statistical significance was determined using a two-tailed unpaired Student's t-test.

**Supplementary Figure 6.** **S100a9 inhibition reduces inflammation at day 7 after IRI.**

(A) Representative H&E staining of kidney sections from DMSO‑ and TASQ‑treated mice. Scale bars, 50 µm; right, quantification of tubular injury score (n=4). (B) Representative images and quantification of TUNEL staining (red). (C) Representative immunofluorescence staining of CD8 (red) in kidney sections. Scale bars, 20 µm; right, quantification of mean fluorescence intensity (MFI) of CD8 signal (n=4). (D) IL-1β and (E) IL-6 mRNA expression in two groups (n=4). (F, G) Flow‑cytometric analysis of renal CD8⁺ T cells and IFN-γ-producing cells among the CD8⁺ T cell population in the kidneys from DMSO‑ and TASQ‑treated mice (n=4). For all quantitative data, statistical significance between the two treatment groups (DMSO vs. TASQ) was determined using a two-tailed unpaired Student's t-test. Data are presented as mean ± SD.

**Supplementary Figure 7. S100a9 inhibition reduces renal injury in cisplatin-induced AKI**

(A) CD63-PAGFP^ITGAM-KI^ mice were subjected to intraperitoneal injection of cisplatin and treated with either the S100a9 inhibitor TASQ or a control (DMSO) via oral gavage, as depicted in the schematic. (B) Representative H&E staining of kidney sections and quantification of tubular injury score (n=4). (C) Representative images and quantification of TUNEL staining (red). (D) Representative immunofluorescence staining for CD8 in kidney sections with quantification of mean fluorescence intensity (MFI). (E, F) IL-1β and IL-6 mRNA expression in kidney tissue from both treatment groups. (G, H) Flow cytometric analysis of the frequency of total renal CD8⁺ T cells and IFN-γ-producing CD8⁺ T cells. For all quantitative data, statistical significance between the two groups was determined using a two-tailed unpaired Student's t-test. Data are presented as mean ± SD.

**Supplementary Figure 8. ITGAM^-^ sEV^IRI^ are not internalized by CD8^+^ T cells**

Time‑course imaging frames (T0–T8) showing ITGAM^-^ sEV^IRI^ monitored without cellular internalization. The top panel (labeled T2') presents a time sequence over 9.11 seconds. The bottom panel display frames from the extended observation period at T0, T4, T6 and T8.
