# Supplementary material for: ITGAM+ Extracellular Vesicles Trigger CD8+ T Cell Activation via S100a9‐Mediated Metabolic Reprogramming in Acute Kidney Injury
Source: J Extracell Vesicles. 2026 Nov 5;15(11):e70404. doi: 10.1002/jev2.70404 (PMC13739760; doi:10.1002/jev2.70404)
Supplement: Supplementary file 2 [file JEV2-15-e70404-s003.docx]

| **gene name** | **protein name** | **Uniprot** | **Protein class** |
| --- | --- | --- | --- |
| ABCG2 | ATP binding cassette subfamily G member 2 | Q9UNQ0 | Cancer-related genes, CD markers, Enzymes, Metabolic proteins, Predicted intracellular proteins, Predicted membrane proteins, Transporters |
| ADAM10 | Disintegrin and metalloproteinase domain-containing protein 10 | O14672 | Cancer-related genes, CD markers, Disease related genes, Enzymes, Plasma proteins, Potential drug targets, Predicted intracellular proteins, Predicted membrane proteins, Transporters |
| ADGRG1 | adhesion G protein-coupled receptor G1 | Q9Y653 | Disease related genes, G-protein coupled receptors, Human disease related genes, Potential drug targets, Predicted intracellular proteins, Predicted membrane proteins, Predicted secreted proteins |
| ADIPOQ | adiponectin | Q15848 | Candidate cardiovascular disease genes, Disease related genes, Human disease related genes, Plasma proteins, Potential drug targets, Predicted secreted proteins, Transporters |
| ALCAM | activated leukocyte cell adhesion molecule | Q13740 | Cancer-related genes, CD markers, Plasma proteins, Predicted membrane proteins, Predicted secreted proteins, Transporters |
| ALDH1A1 | Retinal dehydrogenase 1 | P00352 | Enzymes, Metabolic proteins, Plasma proteins, Predicted intracellular proteins |
| AMIGO1 | Amphoterin-induced protein 1 | Q86WK6 | Predicted membrane proteins, Transporters |
| AMIGO2 | Amphoterin-induced protein 2 | Q86SJ2 | Predicted membrane proteins |
| ANPEP | alanyl aminopeptidase, membrane | P15144 | CD markers, Enzymes, Metabolic proteins, Plasma proteins, Predicted membrane proteins |
| ANXA1 | annexin A1 | P04083 | Cancer-related genes, FDA approved drug targets, Plasma proteins, Predicted intracellular proteins, Predicted secreted proteins, Transporters |
| AXL | AXL receptor tyrosine kinase receptor UFO | P30530 | Disease related genes, Enzymes, FDA approved drug targets, Metabolic proteins, Plasma proteins, Predicted membrane proteins |
| BCAM | basal cell adhesion molecule | P50895 | Blood group antigen proteins, CD markers, Plasma proteins, Predicted intracellular proteins, Predicted membrane proteins |
| BOC | BOC cell adhesion associated, oncogene regulated | Q9BWV1 | Predicted intracellular proteins, Predicted membrane proteins |
| CADM3 | Cell adhesion molecule 3 | Q8N126 | Predicted intracellular proteins, Predicted membrane proteins |
| CADM4 | cell adhesion molecule 4 | Q8NFZ8 | Predicted membrane proteins |
| CAV1 | Caveolin-1 | Q03135 | Cancer-related genes, Disease related genes, Human disease related genes, Potential drug targets, Predicted intracellular proteins, Predicted membrane proteins, Transporters |
| CCR2 | C-C motif chemokine receptor 2 | P41597 | CD markers, G-protein coupled receptors, Human disease related genes, Predicted membrane proteins |
| CCR6 | C-C motif chemokine receptor 6 | P51684 | CD markers, G-protein coupled receptors, Human disease related genes, Predicted membrane proteins |
| CD151 | CD151 antigen | P48509 | Blood group antigen proteins, CD markers, Disease related genes, Human disease related genes, Potential drug targets, Predicted membrane proteins, Transporters |
| CD20 | CD20 | P11836 | CD markers, Disease related genes, FDA approved drug targets, Human disease related genes, Predicted intracellular proteins, Predicted membrane proteins, Transporters |
| CD22 | CD22 molecule | P20273 | CD markers, FDA approved drug targets, Plasma proteins, Predicted intracellular proteins, Predicted membrane proteins |
| CD24 | Signal transducer CD24 | P25063 | Cancer-related genes, CD markers, Disease related genes, Predicted intracellular proteins |
| CD26 | CD26/Dipeptidyl peptidase 4 | P27487 | CD markers, Enzymes, FDA approved drug targets, Plasma proteins, Predicted intracellular proteins, Predicted secreted proteins |
| CD27 | CD27 molecule | P26842 | Cancer-related genes, CD markers, Disease related genes, Plasma proteins, Predicted membrane proteins |
| CD274 | CD274 molecule | Q9NZQ7 | Cancer-related genes, CD markers, Disease related genes, FDA approved drug targets, Predicted membrane proteins |
| CD33 | CD33 molecule | P20138 | CD markers, FDA approved drug targets, Plasma proteins, Predicted membrane proteins |
| CD36 | CD36/Platelet glycoprotein 4 | P16671 | Cancer-related genes, CD markers, Disease related genes, Human disease related genes, Metabolic proteins, Plasma proteins, Potential drug targets, Predicted membrane proteins, Transporters |
| CD44 | CD44 antigen | P16070 | Blood group antigen proteins, Cancer-related genes, CD markers, FDA approved drug targets, Plasma proteins, Predicted intracellular proteins, Predicted membrane proteins, Predicted secreted proteins |
| CD63 | CD63 antigen | P08962 | CD markers, Plasma proteins, Predicted membrane proteins, Transporters |
| CD81 | CD81 antigen | P60033 | CD markers, Disease related genes, Human disease related genes, Plasma proteins, Potential drug targets, Predicted membrane proteins, Transporters |
| CD9 | CD9 antigen | P21926 | Cancer-related genes, CD markers, Plasma proteins, Predicted membrane proteins, Transporters |
| CDCP1 | CUB domain-containing protein 1 | Q9H5V8 | CD markers, Predicted intracellular proteins, Predicted membrane proteins |
| CDH1 | cadherin 1 | P12830 | Cancer-related genes, CD markers, Disease related genes, Human disease related genes, Plasma proteins, Predicted membrane proteins |
| CDH11 | cadherin 11 | P55287 | Cancer-related genes, Disease related genes, Human disease related genes, Predicted intracellular proteins, Predicted membrane proteins |
| CDH12 | cadherin 12 | P55289 | Plasma proteins, Predicted membrane proteins |
| CDH13 | cadherin 13 | P55290 | Plasma proteins, Predicted intracellular proteins |
| CDH15 | Cadherin-15 | P55291 | Disease related genes, Human disease related genes, Predicted membrane proteins |
| CDH17 | Cadherin-17 | Q12864 | Cancer-related genes, Plasma proteins, Predicted intracellular proteins, Predicted membrane proteins |
| CDH2 | Cadherin-2 | P19022 | CD markers, Human disease related genes, Plasma proteins, Predicted intracellular proteins, Predicted membrane proteins |
| CDH3 | cadherin 3 | P22223 | Disease related genes, Human disease related genes, Predicted intracellular proteins, Predicted membrane proteins |
| CDH4 | cadherin 4 | P55283 | Predicted membrane proteins |
| CDH5 | cadherin 5 | P33151 | Cancer-related genes, CD markers, Plasma proteins, Predicted intracellular proteins, Predicted membrane proteins |
| CDH6 | cadherin 6 | P55285 | Plasma proteins, Predicted membrane proteins |
| CDHR5 | Cadherin-related family member 5 | Q9HBB8 | Plasma proteins, Predicted intracellular proteins, Predicted membrane proteins |
| CDON | Cell adhesion molecule-related/down-regulated by oncogenes | Q4KMG0 | Disease related genes, Human disease related genes, Predicted intracellular proteins, Predicted membrane proteins |
| CEACAM1 | CEA cell adhesion molecule 1 | P13688 | CD markers, Plasma proteins, Predicted intracellular proteins, Predicted membrane proteins |
| CEACAM3 | CEA cell adhesion molecule 3 | P40198 | CD markers, Predicted intracellular proteins, Predicted membrane proteins |
| CEACAM4 | Carcinoembryonic antigen-related cell adhesion molecule 4 | O75871 | Predicted membrane proteins |
| CEACAM5 | CEA cell adhesion molecule 5 | P06731 | Cancer-related genes, CD markers, Plasma proteins, Predicted intracellular proteins |
| CEACAM6 | Carcinoembryonic antigen-related cell adhesion molecule 6 | P40199 | Cancer-related genes, CD markers, Predicted intracellular proteins, Predicted membrane proteins |
| CEACAM7 | CEA cell adhesion molecule 7 | Q14002 | Predicted intracellular proteins, Predicted membrane proteins |
| CEACAM8 | Carcinoembryonic antigen-related cell adhesion molecule 8 | P31997 | CD markers, Plasma proteins, Predicted intracellular proteins |
| CLDN1 | claudin 1 | O95832 | Disease related genes, Human disease related genes, Potential drug targets, Predicted membrane proteins, Transporters |
| CLDN10 | claudin 10 | P78369 | Disease related genes, Human disease related genes, Predicted intracellular proteins, Predicted membrane proteins |
| CLDN11 | claudin 11 | O75508 | Predicted membrane proteins |
| CLDN12 | claudin 12 | P56749 | Predicted membrane proteins |
| CLDN17 | claudin 17 | P56750 | Predicted membrane proteins, Transporters |
| CLDN19 | Claudin-19 | Q8N6F1 | Disease related genes, Human disease related genes, Potential drug targets, Predicted membrane proteins, Transporters |
| CLDN3 | claudin 3 | O15551 | Cancer-related genes, Disease related genes, Human disease related genes, Potential drug targets, Predicted membrane proteins, Transporters |
| CLDN4 | Claudin-4 | O14493 | Cancer-related genes, Disease related genes, Human disease related genes, Potential drug targets, Predicted membrane proteins, Transporters |
| CLDN6 | claudin 6 | P56747 | Predicted intracellular proteins, Predicted membrane proteins |
| CLDN8 | claudin 8 | P56748 | Predicted membrane proteins, Transporters |
| CLEC1B | C-type lectin domain family 1 member B | Q9P126 | Predicted intracellular proteins, Predicted membrane proteins |
| CLEC2A | C-type lectin domain family 2 member A | Q6UVW9 | Predicted membrane proteins |
| CLEC5A | C-type lectin domain containing 5A | Q9NY25 | Predicted intracellular proteins, Predicted membrane proteins |
| CLMP | CXADR like membrane protein | Q9H6B4 | Disease related genes, Human disease related genes, Predicted membrane proteins |
| CLSTN1 | Calsyntenin-1 | O94985 | Cancer-related genes, Plasma proteins, Predicted membrane proteins |
| CPM | carboxypeptidase M | P14384 | Enzymes, Plasma proteins, Predicted intracellular proteins, Predicted membrane proteins |
| CSPG4 | chondroitin sulfate proteoglycan 4 | Q6UVK1 | Predicted membrane proteins |
| CTLA4 | Cytotoxic T-lymphocyte protein 4 | P16410 | CD markers, Disease related genes, FDA approved drug targets, Human disease related genes, Predicted intracellular proteins, Predicted membrane proteins |
| CXADR | CXADR Ig-like cell adhesion molecule | P78310 | Predicted membrane proteins, Predicted secreted proteins, Transporters |
| CXCL16 | C-X-C motif chemokine ligand 16 | Q9H2A7 | Predicted intracellular proteins, Predicted membrane proteins, Predicted secreted proteins, Transporters |
| CXCL8 | C-X-C motif chemokine ligand 8 | P10145 | Cancer-related genes, Candidate cardiovascular disease genes, Plasma proteins, Predicted secreted proteins |
| CXCR4 | C-X-C motif chemokine receptor 4 | P61073 | Cancer-related genes, CD markers, Disease related genes, FDA approved drug targets, G-protein coupled receptors, Human disease related genes, Predicted membrane proteins, Transporters |
| DSC1 | desmocollin 1 | Q08554 | Plasma proteins, Predicted membrane proteins |
| DSC2 | desmocollin 2 | Q02487 | Disease related genes, Human disease related genes, Plasma proteins, Predicted membrane proteins |
| DSC3 | Desmocollin-3 | Q14574 | Disease related genes, Human disease related genes, Plasma proteins, Predicted membrane proteins |
| DSCAM | DS cell adhesion molecule | O60469 | Plasma proteins, Predicted membrane proteins |
| DSCAML1 | DS cell adhesion molecule like 1 | Q8TD84 | Predicted intracellular proteins, Predicted membrane proteins |
| DSG1 | Desmoglein-1 | Q02413 | Disease related genes, Human disease related genes, Plasma proteins, Predicted membrane proteins |
| DSG2 | Desmoglein-2 | Q14126 | Disease related genes, Human disease related genes, Plasma proteins, Predicted intracellular proteins, Predicted membrane proteins |
| DSG3 | Desmoglein-3 | P32926 | Plasma proteins, Predicted membrane proteins |
| DSG4 | Desmoglein-4 | Q86SJ6 | Disease related genes, Human disease related genes, Predicted membrane proteins |
| EFNB2 | ephrin B2 | P52799 | Cancer-related genes, Plasma proteins, Predicted membrane proteins |
| EGFR | Epidermal growth factor receptor | P00533 | Cancer-related genes, Disease related genes, Enzymes, FDA approved drug targets, Human disease related genes, Metabolic proteins, Plasma proteins, Predicted intracellular proteins, Predicted membrane proteins, Predicted secreted proteins, RAS pathway related proteins |
| EMCN | Endomucin | Q9ULC0 | Predicted intracellular proteins, Predicted membrane proteins |
| EPCAM | Epithelial cell adhesion molecule | P16422 | Cancer-related genes, CD markers, Disease related genes, FDA approved drug targets, Human disease related genes, Plasma proteins, Predicted intracellular proteins, Predicted membrane proteins |
| EPHA2 | EPH receptor A2 | P29317 | Cancer-related genes, Disease related genes, Enzymes, FDA approved drug targets, Human disease related genes, Metabolic proteins, Predicted membrane proteins, RAS pathway related proteins |
| ERBB2 | Receptor tyrosine-protein kinase erbB-2 | P04626 | Cancer-related genes, CD markers, Disease related genes, Enzymes, FDA approved drug targets, Human disease related genes, Metabolic proteins, Plasma proteins, Predicted intracellular proteins, Predicted membrane proteins |
| ESAM | Endothelial cell-selective adhesion molecule | Q96AP7 | Plasma proteins, Predicted membrane proteins |
| F11R | F11 receptor | Q9Y624 | CD markers, Plasma proteins, Predicted membrane proteins |
| FN1 | fibronectin 1 | P02751 | Cancer-related genes, Candidate cardiovascular disease genes, Disease related genes, FDA approved drug targets, Human disease related genes, Plasma proteins, Predicted intracellular proteins, Predicted secreted proteins |
| FOLH1 | Glutamate carboxypeptidase 2 | Q04609 | Cancer-related genes, Enzymes, FDA approved drug targets, Plasma proteins, Predicted intracellular proteins, Predicted membrane proteins |
| GPA33 | Cell surface A33 antigen | Q99795 | Cancer-related genes, Plasma proteins, Predicted intracellular proteins, Predicted membrane proteins |
| GPC1 | Glypican-1 | P35052 | Disease related genes, Predicted intracellular proteins, Predicted membrane proteins |
| GSN | gelsolin | P06396 | Cancer-related genes, Disease related genes, Human disease related genes, Plasma proteins, Predicted intracellular proteins, Predicted secreted proteins |
| HEPACAM | hepatic and glial cell adhesion molecule | Q14CZ8 | Disease related genes, Human disease related genes, Potential drug targets, Predicted membrane proteins, Transporters |
| HLA-A | HLA class I histocompatibility antigen | P04439 | Cancer-related genes, Disease related genes, Human disease related genes, Plasma proteins, Potential drug targets, Predicted intracellular proteins, Predicted membrane proteins, Transporters |
| HLA-DRA | HLA class II histocompatibility antigen | P01903 | Plasma proteins, Predicted membrane proteins |
| ICAM1 | Intercellular adhesion molecule 1 | P05362 | Cancer-related genes, Candidate cardiovascular disease genes, CD markers, FDA approved drug targets, Human disease related genes, Plasma proteins, Predicted intracellular proteins, Predicted membrane proteins, Transporters |
| ICAM2 | intercellular adhesion molecule 2 | P13598 | CD markers, Plasma proteins, Predicted intracellular proteins, Predicted membrane proteins |
| ICAM3 | Intercellular adhesion molecule 3 | P32942 | CD markers, Plasma proteins, Predicted intracellular proteins, Predicted membrane proteins |
| ICAM4 | intercellular adhesion molecule 4 | Q14773 | Blood group antigen proteins, CD markers, Predicted intracellular proteins, Predicted membrane proteins |
| IL1RAPL1 | interleukin 1 receptor accessory protein like 1 | Q9NZN1 | Disease related genes, Enzymes, Human disease related genes, Plasma proteins, Potential drug targets, Predicted membrane proteins |
| IL6 | Interleukin-6 | P05231 | Cancer-related genes, Candidate cardiovascular disease genes, Disease related genes, FDA approved drug targets, Human disease related genes, Plasma proteins, Predicted intracellular proteins, Predicted secreted proteins |
| ILK | integrin linked kinase | Q13418 | Cancer-related genes, Enzymes, Metabolic proteins, Plasma proteins, Predicted intracellular proteins |
| ITGA1 | Integrin alpha-1 | P56199 | Cancer-related genes, CD markers, Plasma proteins, Predicted membrane proteins, Transporters |
| ITGA11 | Integrin alpha-11 | Q9UKX5 | Predicted membrane proteins |
| ITGA2 | Integrin alpha-2 | P17301 | Cancer-related genes, CD markers, Human disease related genes, Plasma proteins, Predicted membrane proteins |
| ITGA2B | Integrin alpha-2 beta | P08514 | Cancer-related genes, Candidate cardiovascular disease genes, CD markers, Disease related genes, FDA approved drug targets, Human disease related genes, Plasma proteins, Predicted intracellular proteins, Predicted membrane proteins |
| ITGA3 | Integrin alpha-3 | P26006 | Cancer-related genes, CD markers, Disease related genes, Human disease related genes, Predicted intracellular proteins, Predicted membrane proteins |
| ITGA4 | Integrin alpha-4 | P13612 | Cancer-related genes, CD markers, FDA approved drug targets, Plasma proteins, Predicted intracellular proteins, Predicted membrane proteins |
| ITGA4B7 | Integrin alpha 4 beta 7 |  |  |
| ITGA5 | Integrin alpha-5 | P08648 | Cancer-related genes, CD markers, Plasma proteins, Predicted membrane proteins |
| ITGA6 | Integrin alpha-6 | P23229 | Cancer-related genes, CD markers, Disease related genes, Human disease related genes, Plasma proteins, Potential drug targets, Predicted intracellular proteins, Predicted membrane proteins, Transporters |
| ITGA8 | Integrin alpha-8 | P53708 | Disease related genes, Human disease related genes, Plasma proteins, Predicted membrane proteins |
| ITGA9 | Integrin alpha-9 | Q13797 | Human disease related genes, Predicted intracellular proteins, Predicted membrane proteins |
| ITGAL | Integrin alpha-L | P20701 | CD markers, FDA approved drug targets, Predicted intracellular proteins, Predicted membrane proteins, Transporters |
| ITGAM | integrin subunit alpha M | P11215 | Cancer-related genes, CD markers, Disease related genes, Predicted intracellular proteins, Predicted membrane proteins |
| ITGAV | Integrin alpha-V | P06756 | Cancer-related genes, CD markers, FDA approved drug targets, Predicted intracellular proteins, Predicted membrane proteins, Transporters |
| ITGAX | Integrin alpha-X | P20702 | CD markers, FDA approved drug targets, Predicted intracellular proteins, Predicted membrane proteins |
| ITGB1 | integrin subunit beta 1 | P05556 | Cancer-related genes, CD markers, FDA approved drug targets, Plasma proteins, Predicted intracellular proteins, Predicted membrane proteins, Transporters |
| ITGB1 | Integrin beta-1 | P05556 | Cancer-related genes, CD markers, FDA approved drug targets, Plasma proteins, Predicted intracellular proteins, Predicted membrane proteins, Transporters |
| ITGB2 | Integrin beta-2 | P05107 | CD markers, Disease related genes, Human disease related genes, Plasma proteins, Predicted intracellular proteins, Predicted membrane proteins |
| ITGB3 | integrin subunit beta 3 | P05106 | Cancer-related genes, CD markers, Disease related genes, FDA approved drug targets, Human disease related genes, Plasma proteins, Predicted intracellular proteins, Predicted membrane proteins |
| ITGB4 | Integrin beta-4 | P16144 | Cancer-related genes, CD markers, Disease related genes, Human disease related genes, Plasma proteins, Predicted intracellular proteins, Predicted membrane proteins |
| ITGB5 | Integrin beta-5 | P18084 | Cancer-related genes, Plasma proteins, Predicted intracellular proteins, Predicted membrane proteins |
| ITGB6 | Integrin beta-6 | P18564 | Cancer-related genes, Disease related genes, Human disease related genes, Plasma proteins, Predicted membrane proteins |
| ITGB7 | Integrin beta-7 | P26010 | Cancer-related genes, FDA approved drug targets, Plasma proteins, Predicted intracellular proteins, Predicted membrane proteins |
| ITGB8 | Integrin beta-8 | P26012 | Cancer-related genes, Predicted membrane proteins |
| JAM2 | Junctional adhesion molecule B, JAM-B | P57087 | CD markers, Disease related genes, Human disease related genes, Predicted membrane proteins |
| JAM3 | Junctional adhesion molecule C | Q9BX67 | Disease related genes, Human disease related genes, Predicted intracellular proteins, Predicted membrane proteins, Predicted secreted proteins |
| JAML | junction adhesion molecule like | Q86YT9 | Predicted intracellular proteins, Predicted membrane proteins |
| KIT | KIT proto-oncogene, receptor tyrosine kinase | P10721 | Cancer-related genes, CD markers, Disease related genes, Enzymes, FDA approved drug targets, Human disease related genes, Metabolic proteins, Plasma proteins, Predicted membrane proteins, RAS pathway related proteins |
| L1CAM | Cell Adhesion Molecule L1 | P32004 | CD markers, Disease related genes, Human disease related genes, Metabolic proteins, Plasma proteins, Predicted intracellular proteins, Predicted membrane proteins |
| LAG3 | lymphocyte activating 3 | P18627 | CD markers, Predicted intracellular proteins, Predicted membrane proteins |
| LAMP1 | lysosomal associated membrane protein 1 | P11279 | CD markers, Plasma proteins, Predicted membrane proteins, Transporters |
| LAMP2 | Lysosome-associated membrane glycoprotein 2 | P13473 | CD markers, Disease related genes, Human disease related genes, Plasma proteins, Potential drug targets, Predicted membrane proteins, Transporters |
| LGALS9 | galectin 9 | O00182 | Predicted intracellular proteins, Predicted secreted proteins |
| LGR5 | leucine rich repeat containing G protein-coupled receptor 5 | O75473 | G-protein coupled receptors, Predicted membrane proteins |
| LYN | Tyrosine-protein kinase Lyn | P07948 | Cancer-related genes, Disease related genes, Enzymes, Metabolic proteins, Potential drug targets, Predicted intracellular proteins |
| MADCAM1 | Mucosal Addressin Cell Adhesion Molecule 1 | Q13477 | Predicted membrane proteins |
| MCAM | melanoma cell adhesion molecule | P43121 | CD markers, Plasma proteins, Predicted membrane proteins |
| MICA | MHC class I polypeptide-related sequence A | Q29983 | Human disease related genes, Predicted intracellular proteins, Predicted membrane proteins |
| MICB | MHC class I polypeptide-related sequence B | Q29980 | Disease related genes, Predicted membrane proteins |
| MMP9 | matrix metallopeptidase 9 | P14780 | Cancer-related genes, Candidate cardiovascular disease genes, Disease related genes, Enzymes, FDA approved drug targets, Human disease related genes, Plasma proteins, Predicted secreted proteins |
| MUC1 | mucin 1 | P15941 | Cancer-related genes, CD markers, Disease related genes, Human disease related genes, Predicted intracellular proteins, Predicted membrane proteins, Predicted secreted proteins |
| MUC16 | Mucin-16(Ovarian carcinoma antigen CA125) | Q8WXI7 | Plasma proteins, Predicted membrane proteins, Predicted secreted proteins |
| MUC19 | Mucin-19 | [Q7Z5P9](https://www.uniprot.org/uniprot/Q7Z5P9) | Predicted intracellular proteins |
| MUC4 | mucin 4, cell surface associated | Q99102 | Predicted membrane proteins, Predicted secreted proteins |
| NCAM1 | neural cell adhesion molecule 1 | P13591 | Cancer-related genes, CD markers, Plasma proteins, Predicted intracellular proteins, Predicted membrane proteins, Predicted secreted proteins |
| NECTIN1 | Nectin-1 | Q15223 | CD markers, Disease related genes, Human disease related genes, Plasma proteins, Predicted membrane proteins, Predicted secreted proteins |
| NECTIN2 | nectin cell adhesion molecule 2 | Q92692 | CD markers, Plasma proteins, Predicted intracellular proteins, Predicted membrane proteins |
| NECTIN3 | nectin cell adhesion molecule 3 | Q9NQS3 | CD markers, Predicted intracellular proteins, Predicted membrane proteins |
| NECTIN4 | nectin cell adhesion molecule 4 | Q96NY8 | Disease related genes, Human disease related genes, Predicted membrane proteins, Predicted secreted proteins |
| NES | nestin | P48681 | Plasma proteins, Predicted intracellular proteins |
| NFASC | neurofascin | O94856 | Disease related genes, Human disease related genes, Plasma proteins, Predicted intracellular proteins, Predicted membrane proteins |
| NGFR | Tumor necrosis factor receptor superfamily member 16 | P08138 | Cancer-related genes, CD markers, Predicted intracellular proteins, Predicted membrane proteins, RAS pathway related proteins |
| NLGN1 | neuroligin 1 | Q8N2Q7 | Disease related genes, Human disease related genes, Potential drug targets, Predicted intracellular proteins, Predicted membrane proteins, Transporters |
| NT5E | 5'-nucleotidase ecto | P21589 | CD markers, Disease related genes, Enzymes, Human disease related genes, Metabolic proteins, Potential drug targets, Predicted intracellular proteins, Predicted membrane proteins |
| NTRK3 | neurotrophic receptor tyrosine kinase 3 | Q16288 | Cancer-related genes, Disease related genes, Enzymes, FDA approved drug targets, Human disease related genes, Metabolic proteins, Plasma proteins, Predicted intracellular proteins, Predicted membrane proteins |
| PAR2 | Proteinase-activated receptor 2 | P55085 | G-protein coupled receptors, Predicted membrane proteins, Transporters |
| PCDH1 | protocadherin 1 | Q08174 | Plasma proteins, Predicted intracellular proteins, Predicted membrane proteins |
| PCDH15 | Protocadherin-15 | Q96QU1 | Disease related genes, Human disease related genes, Plasma proteins, Predicted intracellular proteins, Predicted membrane proteins |
| PCDH17 | protocadherin 17 | O14917 | Predicted intracellular proteins, Predicted membrane proteins |
| PCDH19 | protocadherin 19 | Q8TAB3 | Disease related genes, Human disease related genes, Predicted intracellular proteins, Predicted membrane proteins |
| PCDH8 | protocadherin 8 | O95206 | Plasma proteins, Predicted membrane proteins |
| PCDHA1 | protocadherin alpha 1 | Q9Y5I3 | Predicted membrane proteins, Predicted secreted proteins |
| PCDHGC3 | protocadherin gamma subfamily C, 3 | Q9UN70 | Plasma proteins, Predicted intracellular proteins, Predicted membrane proteins |
| PDCD1 | programmed cell death 1 | Q15116 | CD markers, Disease related genes, FDA approved drug targets, Human disease related genes, Predicted intracellular proteins, Predicted membrane proteins |
| PDPN | podoplanin | Q86YL7 | Predicted membrane proteins |
| PECAM1 | platelet and endothelial cell adhesion molecule 1 | P16284 | Cancer-related genes, Candidate cardiovascular disease genes, CD markers, Plasma proteins, Predicted intracellular proteins, Predicted membrane proteins |
| PLAU | Urokinase-type plasminogen activator | P00749 | Cancer-related genes, Disease related genes, Enzymes, Plasma proteins, Potential drug targets, Predicted intracellular proteins, Predicted secreted proteins |
| PLAUR | plasminogen activator, urokinase receptor | Q03405 | Cancer-related genes, CD markers, FDA approved drug targets, Predicted intracellular proteins, Predicted membrane proteins |
| PLXNB1 | plexin B1 | O43157 | Plasma proteins, Predicted membrane proteins |
| PROM1 | Prominin-1 | O43490 | CD markers, Disease related genes, Human disease related genes, Potential drug targets, Predicted intracellular proteins, Predicted membrane proteins, Transporters |
| PTPRJ | Receptor-type tyrosine-protein phosphatase eta | Q12913 | CD markers, Enzymes, Metabolic proteins, Plasma proteins, Predicted intracellular proteins, Predicted membrane proteins |
| RETN | Resistin | [Q9HD89](https://www.uniprot.org/uniprot/Q9HD89) | Candidate cardiovascular disease genes, Plasma proteins, Predicted secreted proteins |
| SELE | E-selectin | P16581 | Cancer-related genes, Candidate cardiovascular disease genes, CD markers, Plasma proteins, Predicted intracellular proteins, Predicted membrane proteins |
| SELL | L-selectin | P14151 | Cancer-related genes, Candidate cardiovascular disease genes, CD markers, Plasma proteins, Predicted intracellular proteins, Predicted membrane proteins |
| SIGLEC1 | sialic acid binding Ig like lectin 1 | Q9BZZ2 | CD markers, Predicted intracellular proteins, Predicted membrane proteins |
| SIGLEC10 | sialic acid binding Ig like lectin 10 | Q96LC7 | Predicted intracellular proteins, Predicted membrane proteins |
| SIGLEC11 | sialic acid binding Ig like lectin 11 | Q96RL6 | Plasma proteins, Predicted membrane proteins |
| SIGLEC14 | sialic acid binding Ig like lectin 14 | Q08ET2 | Plasma proteins, Predicted membrane proteins |
| SIGLEC5 | Siglec-5 | O15389 | CD markers, Predicted membrane proteins |
| SIGLEC6 | sialic acid binding Ig like lectin 6 | O43699 | CD markers, Predicted membrane proteins, Predicted secreted proteins |
| SIGLEC7 | sialic acid binding Ig like lectin 7 | Q9Y286 | CD markers, Predicted intracellular proteins, Predicted membrane proteins |
| SIGLEC8 | Siglec-8 | Q9NYZ4 | CD markers, Plasma proteins, Predicted membrane proteins |
| SIGLEC9 | sialic acid binding Ig like lectin 9 | Q9Y336 | Predicted membrane proteins |
| TACSTD2 | Tumor-associated calcium signal transducer 2 | P09758 | Disease related genes, FDA approved drug targets, Human disease related genes, Predicted membrane proteins |
| TENM1 | Teneurin-1 | Q9UKZ4 | Predicted membrane proteins, Transporters |
| TENM2 | Teneurin-2 | Q9NT68 | Predicted membrane proteins, Transporters |
| TENM4 | Teneurin-4 | Q6N022 | Disease related genes, Human disease related genes, Predicted intracellular proteins, Predicted membrane proteins |
| THY1 | CD90/Thy-1 membrane glycoprotein | P04216 | CD markers, Plasma proteins, Predicted intracellular proteins, Predicted membrane proteins |
| TICAM2 | toll like receptor adaptor molecule 2 | Q86XR7 | Predicted intracellular proteins |
| TIM3 | Hepatitis A virus cellular receptor 2 | Q8TDQ0 | CD markers, Disease related genes, Predicted intracellular proteins, Predicted membrane proteins |
| TIMP2 | TIMP metallopeptidase inhibitor 2 | P16035 | Cancer-related genes, Candidate cardiovascular disease genes, Plasma proteins, Predicted intracellular proteins, Predicted secreted proteins |
| TMEM204 | Transmembrane protein 204 | Q9BSN7 | Predicted membrane proteins, Transporters |
| TPBG | Trophoblast glycoprotein | Q13641 | Predicted membrane proteins |
| ULBP1 | UL16 binding protein 1 | Q9BZM6 | Predicted membrane proteins |
| ULBP2 | UL16 binding protein 2 | Q9BZM5 | Predicted membrane proteins, Predicted secreted proteins |
| ULBP3 | UL16-binding protein 3 | Q9BZM4 | Predicted intracellular proteins |
| VCAM1 | Vascular cell adhesion protein 1 | P19320 | Cancer-related genes, CD markers, Plasma proteins, Predicted membrane proteins |
| TNFRSF9 | TNF receptor superfamily member 9 | Q07011 | Cancer-related genes, CD markers, Predicted intracellular proteins, Predicted membrane proteins |
| AIF1 | allograft inflammatory factor 1 | P55008 | Predicted intracellular proteins |
| HAVCR1 | hepatitis A virus cellular receptor 1 | Q96D42 | CD markers, Predicted intracellular proteins, Predicted membrane proteins |
| VEGFA | vascular endothelial growth factor A | P15692 | Cancer-related genes, Candidate cardiovascular disease genes, Disease related genes, FDA approved drug targets, Human disease related genes, Plasma proteins, Predicted intracellular proteins, Predicted secreted proteins, RAS pathway related proteins |
| APOE | apolipoprotein E | P02649 | Cancer-related genes, Candidate cardiovascular disease genes, Disease related genes, Human disease related genes, Metabolic proteins, Plasma proteins, Predicted secreted proteins |
| C9 | complement C9 | P02748 | Disease related genes, Human disease related genes, Plasma proteins, Predicted secreted proteins |
| CCR4 | C-C motif chemokine receptor 4 | P51679 | CD markers, FDA approved drug targets, G-protein coupled receptors, Predicted membrane proteins |
| CD14 | CD14 molecule | P08571 | Cancer-related genes, CD markers, Human disease related genes, Plasma proteins, Predicted secreted proteins, Transporters |
| CD163 | CD163 molecule | Q86VB7 | CD markers, Plasma proteins, Predicted intracellular proteins, Predicted membrane proteins, Predicted secreted proteins |
| CD19 | CD19 molecule | P15391 | CD markers, Disease related genes, FDA approved drug targets, Human disease related genes, Predicted membrane proteins |
| CD1C | CD1c molecule | P29017 | CD markers, Predicted intracellular proteins, Predicted membrane proteins |
| CD28 | CD28 molecule | P10747 | CD markers, Predicted membrane proteins |
| CD38 | CD38 molecule | P28907 | Cancer-related genes, CD markers, Enzymes, Metabolic proteins, Predicted intracellular proteins |
| CD3E | CD3e molecule | P07766 | CD markers, Disease related genes, FDA approved drug targets, Human disease related genes, Predicted membrane proteins, Transporters |
| CD4 | CD4 molecule | P01730 | CD markers, FDA approved drug targets, Predicted intracellular proteins, Predicted membrane proteins, Transporters |
| CD40 | CD40 molecule | P25942 | Cancer-related genes, CD markers, Disease related genes, Human disease related genes, Predicted membrane proteins, Predicted secreted proteins |
| CD5 | CD5 molecule | P06127 | CD markers, Predicted intracellular proteins, Predicted membrane proteins |
| CD68 | CD68 molecule | P34810 | CD markers, Plasma proteins, Predicted intracellular proteins, Predicted membrane proteins |
| CD80 | CD80 molecule | P33681 | CD markers, FDA approved drug targets, Predicted intracellular proteins, Predicted membrane proteins |
| CD86 | CD86 molecule | P42081 | CD markers, FDA approved drug targets, Predicted intracellular proteins, Predicted membrane proteins |
| CD8A | CD8a molecule | P01732 | CD markers, Disease related genes, Human disease related genes, Predicted intracellular proteins, Predicted membrane proteins |
| CLEC4C | C-type lectin domain family 4 member C | Q8WTT0 | CD markers, Predicted intracellular proteins, Predicted membrane proteins |
| CLEC9A | C-type lectin domain containing 9A | Q6UXN8 | CD markers, Predicted membrane proteins |
| CR1 | complement C3b/C4b receptor 1 | P17927 | Blood group antigen proteins, CD markers, Plasma proteins, Predicted membrane proteins |
| CR2 | complement C3d receptor 2 | P20023 | CD markers, Disease related genes, Human disease related genes, Plasma proteins, Predicted intracellular proteins, Predicted membrane proteins |
| CSF3R | colony stimulating factor 3 receptor | Q99062 | Cancer-related genes, CD markers, Disease related genes, FDA approved drug targets, Human disease related genes, Predicted intracellular proteins, Predicted membrane proteins |
| CUBN | cubilin | O60494 | Disease related genes, Human disease related genes, Metabolic proteins, Predicted intracellular proteins |
| CXCR2 | C-X-C motif chemokine receptor 2 | P25025 | Cancer-related genes, CD markers, G-protein coupled receptors, Predicted intracellular proteins, Predicted membrane proteins |
| EMCN | endomucin | Q9ULC0 | Predicted intracellular proteins, Predicted membrane proteins |
| ENG | endoglin | P17813 | Cancer-related genes, Candidate cardiovascular disease genes, CD markers, Disease related genes, Human disease related genes, Plasma proteins, Predicted membrane proteins |
| FAS | Fas cell surface death receptor | P25445 | Cancer-related genes, Candidate cardiovascular disease genes, CD markers, Disease related genes, Human disease related genes, Metabolic proteins, Predicted membrane proteins, Predicted secreted proteins |
| FCGR1A | Fc gamma receptor Ia | P12314 | CD markers, FDA approved drug targets, Predicted membrane proteins |
| ICOS | inducible T cell costimulator | Q9Y6W8 | CD markers, Disease related genes, Human disease related genes, Predicted membrane proteins |
| IGF2 | insulin like growth factor 2 | P01344 | Cancer-related genes, Disease related genes, Human disease related genes, Plasma proteins, Predicted membrane proteins, Predicted secreted proteins, RAS pathway related proteins |
| IL2RA | interleukin 2 receptor subunit alpha | P01589 | Cancer-related genes, CD markers, Disease related genes, FDA approved drug targets, Human disease related genes, Plasma proteins, Predicted intracellular proteins, Predicted membrane proteins |
| IL2RB | interleukin 2 receptor subunit beta | P14784 | Cancer-related genes, CD markers, Disease related genes, FDA approved drug targets, Predicted intracellular proteins, Predicted membrane proteins, Transporters |
| IL3RA | interleukin 3 receptor subunit alpha | P26951 | CD markers, FDA approved drug targets, Predicted intracellular proteins, Predicted membrane proteins |
| IL7R | interleukin 7 receptor | P16871 | Cancer-related genes, CD markers, Disease related genes, Human disease related genes, Predicted intracellular proteins, Predicted membrane proteins |
| KLRD1 | killer cell lectin like receptor D1 | Q13241 | CD markers, Predicted intracellular proteins, Predicted membrane proteins |
| LRP2 | LDL receptor related protein 2 | P98164 | Disease related genes, Human disease related genes, Metabolic proteins, Plasma proteins, Potential drug targets, Predicted intracellular proteins, Predicted membrane proteins, Transporters |
| MPO | myeloperoxidase | P05164 | Cancer-related genes, Candidate cardiovascular disease genes, Disease related genes, Enzymes, Human disease related genes, Metabolic proteins, Plasma proteins, Potential drug targets, Predicted intracellular proteins |
| MRC1 | mannose receptor C-type 1 | P22897 | CD markers, FDA approved drug targets, Plasma proteins, Predicted membrane proteins |
| NCR1 | natural cytotoxicity triggering receptor 1 | O76036 | CD markers, Predicted membrane proteins |
| NPHS1 | NPHS1 adhesion molecule, nephrin | O60500 | Disease related genes, Human disease related genes, Plasma proteins, Potential drug targets, Predicted intracellular proteins, Predicted membrane proteins, Transporters |
| PTPRC | protein tyrosine phosphatase receptor type C | P08575 | Cancer-related genes, CD markers, Disease related genes, Enzymes, Human disease related genes, Plasma proteins, Potential drug targets, Predicted intracellular proteins, Predicted membrane proteins, Transporters |
| SDC1 | syndecan 1 | P18827 | Cancer-related genes, CD markers, Metabolic proteins, Plasma proteins, Predicted intracellular proteins, Predicted membrane proteins, Predicted secreted proteins, Transporters |
| SIRPA | signal regulatory protein alpha | P78324 | CD markers, Plasma proteins, Predicted membrane proteins |
| SLC12A1 | solute carrier family 12 member 1 | Q13621 | Disease related genes, FDA approved drug targets, Human disease related genes, Metabolic proteins, Predicted membrane proteins, Transporters |
| SLC12A3 | solute carrier family 12 member 3 | P55017 | Disease related genes, FDA approved drug targets, Human disease related genes, Metabolic proteins, Plasma proteins, Predicted membrane proteins, Transporters |
| SLC34A1 | solute carrier family 34 member 1 | Q06495 | Disease related genes, Human disease related genes, Metabolic proteins, Plasma proteins, Potential drug targets, Predicted intracellular proteins, Predicted membrane proteins, Transporters |
| SLC5A12 | solute carrier family 5 member 12 | Q1EHB4 | Metabolic proteins, Predicted membrane proteins, Transporters |
| SLC5A2 | solute carrier family 5 member 2 | P31639 | Disease related genes, FDA approved drug targets, Human disease related genes, Metabolic proteins, Predicted membrane proteins, Transporters |
| THBD | thrombomodulin | P07204 | Candidate cardiovascular disease genes, CD markers, Disease related genes, Human disease related genes, Predicted membrane proteins |
| XCR1 | X-C motif chemokine receptor 1 | P46094 | G-protein coupled receptors, Human disease related genes, Predicted intracellular proteins, Predicted membrane proteins |
| TMEM119 | transmembrane protein 119 | Q4V9L6 | Predicted intracellular proteins, Predicted membrane proteins, Transporters |
| ACE2 |  |  |  |
| AQP1 |  |  |  |
| AQP2 |  |  |  |

**Supplementary Table1 Antibody panel used for Proximity Barcoding Assay (PBA).**

|  |  | **Subgroup** | **Marker** |
| --- | --- | --- | --- |
| **Immune  cell** | Innate  immune cell | Monocyte | CD14 |
|  |  | Macrophage | CD86(M1); CD206(M2) |
|  |  | Neutrophil | Ly6G |
|  |  | Natural kill(NK)cell | CD56 |
|  |  | Dentric cell | CD11c |
|  |  | Basophil | CD13 |
|  |  | Mast cell | KIT |
|  | Adaptive  immune cell | T cell | CD4(CD4+T); CD8(CD8+T) |
|  |  | B cell | CD20 |
| **Resident  cell** | Glomerular  cell | Podocyte | NPHS1 |
|  |  | Parietal  epithelial cell | CLDN1 |
|  |  | Endothelial cell | ITGA8 |
|  | Tubular  cell | Proximal  tubular cell | SLC5A2; LRP2; SLC34A1 |
|  |  | Long descending limb | CD40 |
|  |  | Acending  loop of henle | SLC12A1 |
|  |  | Distal convoluted  tubular cell | SLC12A3 |
|  |  | Collecting duct  principal cell | AQP2 |
|  | Fibroblast |  | FN1 |
|  | Progenitor  cell |  | PROM1 |

**Supplementary Table2. Canonical markers for cellular sourcing of extracellular vesicles.**
