# Supplementary material for: ITGAM+ Extracellular Vesicles Trigger CD8+ T Cell Activation via S100a9‐Mediated Metabolic Reprogramming in Acute Kidney Injury
Source: J Extracell Vesicles. 2026 Nov 5;15(11):e70404. doi: 10.1002/jev2.70404 (PMC13739760; doi:10.1002/jev2.70404)
Supplement: Supplementary file 3 [file JEV2-15-e70404-s005.docx]

**Supplementary Methods**

**Tissue Morphology Study**

Kidneys were fixed in 4% paraformaldehyde, paraffin‑embedded, and sectioned at 4μm. To quantify renal tubular injury, kidney sections were examined under a light microscope. The degree of histopathological damage, including tubular dilation, cast formation, brush border loss, and tubular necrosis, was assessed in ten randomly selected, non-overlapping high-power fields (400x) per section. A semiquantitative score was assigned based on the percentage of tubules affected: 0 (no injury), 1 (<25% injured), 2 (25-50% injured), 3 (50-75% injured), and 4 (>75% injured). The final injury score for each animal was the average of the scores from all fields.

**TUNEL Assay**

Tubular cell apoptosis was detected using a One-Step TUNEL Apoptosis Assay Kit (C1089, Beyotime) according to the manufacturer’s protocol. Briefly, permeabilized sections were incubated with the TUNEL reaction mixture to label fragmented DNA, followed by counterstaining with DAPI. All slides were imaged using a fluorescence microscope, and quantification was performed using ImageJ software.

**Immunofluorescence Staining**

Following deparaffinization and rehydration, paraffin-embedded tissue sections underwent antigen retrieval using Tris-EDTA buffer and were then cooled to room temperature. Frozen tissue sections were permeabilized with 0.25% Triton X-100. Both antigen-retrieved paraffin sections and permeabilized frozen sections were blocked with 5% BSA for 30 minutes, incubated with primary antibodies:ITGAM(sc-1186,Santa-Cruz); F4/80(14-480-1-82, Invitrogen);CD11c(14-0114-82, Invitrogen);LY6G(Servicebio,GB11229;CD8(3057173,Invitrogen);LYVE1(ab218535,abcam);S100a9(26992-1-AP, Proteintech);Slc25a12(67467-1-Ig, Proteintech) at 4°C overnight, and subsequently treated with secondary antibodies for 1 hour protected from light. Imaging was finally captured using confocal microscopy. ImageJ was employed for image analysis.

For suspended CD8^+^T cells, cells were fixed in PFA, permeabilized with 0.25% Triton X‑100, blocked with 2% BSA, and stained following the same protocol as above.

**Flow Cytometry**

Cells were first blocked with 5% rat serum at 4°C for 20 minutes, followed by incubation with surface marker antibodies: allophycocyanin(APC) anti-mouse CD8(100712, Biolegend, 0.2mg/ml) at 4°C for 30 minutes. The cells were then permeabilized by adding a permeabilization buffer while vortexing, followed by incubation at room temperature for 40 minutes in the dark. Subsequently, intracellular staining was performed using specific antibodies: phycoerythrin(PE) anti-mouse IFN-γ(505807,Biolegend,0.2mg/ml) or fluorescein isothiocyanate(FITC) anti-mouse IFN-γ(505805,Biolegend,0.5mg/ml) ; Ki67 Monoclonal Antibody (SolA15)-PE(12-5698-82, Invitrogen, 0.2mg/ml) SLC25A12(MA5-57714, Invitrogen, 0.8mg/ml) followed by FITC(BS-0295D, bioss) at 4°C for 30 minutes. After a final wash and centrifugation, the cells were resuspended and acquired on a flow cytometer(BD LSRFortessa X20).

For kidney tissue, a single-cell suspension was prepared using a commercial tissue dissociation kit (RWD, DHGT-10) according to the manufacturer's protocol. The subsequent staining steps followed the same flow cytometry procedure described above.

**Functional Metabolic Assays**

Mitochondria were isolated from CD8⁺ T cells using a Mitochondria Isolation Kit for Cultured Cells (C3601, Beyotime) according to the manufacturer's protocol. Briefly, cells were homogenized using a Dounce homogenizer in reagent-based buffers, followed by differential centrifugation to separate the mitochondrial fraction from the cytosolic fraction.

ATP, NAD⁺/NADH, and Glutamate Assays**:** Intracellular concentrations of ATP (Enhanced ATP Assay Kit, S0027,Beyotime), NAD⁺/NADH (NAD⁺/NADH Assay Kit, S0175, Beyotime), and glutamate (Glutamic Acid Colorimetric Assay Kit, E-BC-K118-S, Elabscience) in CD8⁺ T cell lysates or mitochondrial fractions were measured using the respective commercial kits according to the manufacturers’ instructions. Absorbance or luminescence was read on a plate reader (SpectraMax iD3, Molecular Devices

**Quantitative real-time PCR(qPCR)**

Total RNA was extracted using FreeZol Reagent (Vazyme) and reverse-transcribed into cDNA using 5× HiScript III qRT SuperMix (Vazyme). qPCR was performed on a QuantStudio 3 Real-Time PCR system (Applied Biosystems) using the 2× ChamQ SYBR qPCR Master Mix (Vazyme). The relative expression of target genes was calculated by the 2–ΔΔCT method.

**Western Blot**

For Western blot analysis, kidney tissues, cells, and sEV fractions were lysed using RIPA buffer (Beyotime Biotechnology, Shanghai,China) on ice and subsequently heated at 95°C for 5 minutes. The samples were then separated by 10% SDS-PAGE (Fcmacs, Nanjing) and transferred onto PVDF membranes (Millipore). After transfer, the membranes were incubated with primary antibodies ITGAM(sc-1186,Santa-Cruz), PAGFP (sc-9996, Santa-Cruz), β-actin (m20027xs, abmayt), S100a9(sc-58706, Santa-Cruz), CD9(60232-1-Ig, Proteintech), GM130(ab52649,abcam) followed by a secondary antibody. sEVs purified from identical weights of renal tissue or equal quantities of sEV samples were applied for Western blotting. To ensure analytical accuracy, quantitative data were normalized to sEV-specific markers—CD63(sc-5275,Santa-Cruz)—as loading controls where appropriate.

**Co-Immunoprecipitation (Co-IP)**

To validate the direct interaction between endogenous S100a9 and Slc25a12, ITGAM⁺ sEV-treated primary CD8⁺ T cells were lysed in IP lysis buffer (Beyotime) supplemented with protease and phosphatase inhibitor cocktails. Pre-cleared lysates were incubated overnight at 4 °C with anti-S100a9 antibody (26992-1-AP, Proteintech) or an equal amount of isotype-matched rabbit IgG (30000-0-AP, Proteintech) as a negative control. Immune complexes were then captured with Protein A/G magnetic beads for 2 h at 4 °C with gentle rotation, washed four times with lysis buffer, and eluted by boiling in 1× SDS sample buffer for 5 min at 95 °C. Eluates, together with whole-cell lysate inputs, were resolved by 10% SDS-PAGE and probed for co-precipitated Slc25a12 (67467-1-Ig, Proteintech) and S100a9 (sc-58706, Santa Cruz) by Western blot as described above.

**ELISA**

Concentrations of TNF-α(1217202, Dakewe Biotech Co., Ltd), IFN-γ(1210002, Dakewe Biotech Co., Ltd), and IL-10(1211002, Dakewe Biotech Co., Ltd) were measured using commercial precoated ELISA kits according to the manufacturer’s instructions. Briefly, 100 µL of supernatant was added per well, followed by 50 µL of biotinylated antibody, and incubated at 37 °C for 90 min. After washing, 100 µL of streptavidin-HRP was added and incubated for 30 min at 37 °C. Color development was performed for 15 min and stopped promptly. Absorbance was read at 450 nm with a reference wavelength between 610 nm and 630 nm. The corrected optical density (OD) value was calculated by subtracting the reference value from the measurement at 450 nm.( Specreamax iD3, Molecular Device Co., Ltd).
